# Genome wide identification of the trihelix transcription factors and overexpression of *Gh_A05G2067* (*GT‐2*), a novel gene contributing to increased drought and salt stresses tolerance in cotton

**DOI:** 10.1111/ppl.12920

**Published:** 2019-02-13

**Authors:** Richard O. Magwanga, Joy N. Kirungu, Pu Lu, Xiu Yang, Qi Dong, Xiaoyan Cai, Yanchao Xu, Xingxing Wang, Zhongli Zhou, Yuqing Hou, Regina Nyunja, Stephen G. Agong, Jinping Hua, Baohong Zhang, Kunbo Wang, Fang Liu

**Affiliations:** ^1^ Institute of Cotton Research Chinese Academy of Agricultural Science (ICR, CAAS)/State Key Laboratory of Cotton Biology Anyang 455000 China; ^2^ Jaramogi Oginga Odinga University of Science and Technology School of Biological and Physical Sciences (SBPS), P.O Box 210‐40601, Bondo Kenya; ^3^ China Agricultural University Beijing China; ^4^ North Carolina State University Raleigh North Carolina

## Abstract

We identified 102, 51 and 51 proteins encoded by the trihelix genes in *Gossypium hirsutum*, *Gossypium arboreum* and *Gossypium* *raimondii,* respectively. RNA sequence data and real‐time quantitative polymerase chain reaction analysis showed that *Gh_A05G2067* (*GT‐2*) was highly upregulated under drought and salt stress conditions. Transient expression of *GT‐2‐*green fluorescent protein fusion protein in protoplast showed that *GT‐2* was localized in the nucleus. The overexpression of *GT‐2* conferred an enhanced drought tolerance to cotton, with lower malondialdehyde, hydrogen peroxide contents and higher reactive oxygen scavenging enzyme activities. Moreover, chlorophyll content, relative leaf water content (RLWC), excised leaf water loss (ELWL) and cell membrane stability (CMS) were relatively stable in the *GT‐2*‐overexpressed lines compared to wild‐type (WT). Similarly, stress‐responsive genes *RD29A*, *SOS1*, *ABF4* and *CBL1* were highly upregulated in the *GT‐2‐*overexpressed lines but were significantly downregulated in WT. In addition, the *GT‐2‐*silenced cotton plants exhibited a high level of oxidation injury, due to high levels of oxidant enzymes, in addition to negative effects on CMS, ELWL, RLWC and chlorophyll content. These results mark the foundation for future exploration of the trihelix genes in cotton, with an aim of developing more resilient, versatile and highly tolerant cotton genotypes.

**Abbreviations**
CAT: Catalase
CMS: Cell membrane stability
H_2_O_2_: Hydrogen peroxide
MDA: Malondialdehyde
POD: Peroxidase
RLWC: Relative leaf water content
SLW: Saturated leaf weight
SLW: Saturated leaf weight
SOD: Superoxide dismutase
VIGS: Virus‐induced gene silencing

## Introduction

Abiotic stress factors cause massive losses in various agricultural crops (Rejeb et al. 2014). With the ever‐changing environmental conditions, aggravated due to massive pollution and environmental degradation, drought, salinity, cold among other abiotic stresses, are projected to worsen in the next decade (Suzuki et al. 2014). The various abiotic stresses compromise plants growth and development. Unlike animals, plants bear the full effect of any abiotic stress due to their sessile nature. When abiotic stresses such as drought, salinity, heat, cold among others set in, the plant's internal equilibrium changes, affecting all the biological and physiological activities within the plant (Mott and Parkhurst 1991, Aasamaa and Sõber 2011). Plants have developed various survival strategies to enable them to either reduce the effects caused by the various environmental stresses or to tolerate the impact of the stress (Cramer et al. 2011). The various responses of plants are mainly regulated at the transcriptome levels; plants have evolved various stress‐responsive genes enabling them to tolerate the adverse effects caused by the various stress factors.

The plant's transcription factors (TFs) have been found to have diverse functions in plant growth and in response to abiotic stress factors (Nejat and Mantri 2017). A number of TFs have been found to regulate various plant processes by interacting with *cis*‐promoter elements or with other TFs with primary roles in gene expression (Zhang et al. 2011). Trihelix (TH) TFs are known to bind specifically to GT elements required for the light response in plant (Kaplan‐Levy et al. 2012). The *TH* gene family shares its defining feature with the *MYB* gene family, a trihelical DNA‐binding domain; therefore, the *TH* is believed to evolve from the *MYB* (Fang et al. 2010). Several studies have shown that the trihelix TF gene family, are not only involved in light responsiveness as previously described, but also in enhancing abiotic stress tolerance in various plants. Systemic analysis of the novel trihelix TF gene family in rice showed that they are involved in abiotic stress responses; *OsGT*γ*‐1* was found to be strongly induced by salt stress, slightly induced by drought, cold stresses and ABA treatment (Fang et al. 2010). A trihelix TF of the GT‐4 type has been found to interact with a B3 and AP2/ERF domain‐containing protein (TEM2) in *Arabidopsis thaliana* (Arabidopsis), thus conferring salt stress tolerance (Wang et al. 2014). Another trihelix TF, *AtGT2L,* has been found to have the ability to interact with calcium/calmodulin, thereby conferring cold and salt stress tolerance in plants (Xi et al. 2012). Two types of trihelix TFs isolated from *Glycine max*, *GmGT‐2A* and *GmGT‐2B*, were found to have the ability to confer tolerance to salt, freezing and drought stress in transgenic Arabidopsis plants (Xie et al. 2009).

The trihelix TF family is predominantly found in plants, from previous studies based on determination and characterization of the gene family in rice and Arabidopsis, three subfamilies designated as GTα, GTβ and GTγ were described (Fang et al. 2010). Two years later, Kaplan‐Levy et al. (2012) modified the earlier classification by Fang et al. (2010) and regrouped the genes into 13 subfamilies, designated as GT‐1, GT‐2, GT‐3, GT‐4, GTL1, EDA3, FIP2, PTL, ASIL1, ASIL2, SH4, SIP1 and GTγ (Kaplan‐Levy et al. 2012). To date, there are only 30 and 31 trihelix genes identified in Arabidopsis and rice, respectively. The number of the *TH* genes is relatively low compared to other stress‐responsive TFs such as *bHLH*, *NAC*, *AP2*/*EREBP* and *MYB*, with more than 100 members in Arabidopsis. Despite the important role played by the *TH* genes in plants, the evolutionary and functional information of this gene family in cotton is still unknown. Thus, a functional analysis is needed in order to understand their evolutionary pattern and functions in cotton plant in relation to drought and salt stress tolerance.

Cotton (*Gossypium*) is the most important crop not only for the textile industries but also as an important source of edible oil and protein source for animal feeds (John and Crow 1992). Cotton production is on the rapid decline due to effects caused by environmental stresses (Magwanga et al. 2018a). *Gossypium* is a large genus in the plant kingdom with over 50 species, and *Gossypium hirsutum,* also known as upland cotton, is an allotetraploid species (2n = 4× = 52), with (AD)_1_ genome. It is the most cultivated cotton species globally. *Gossypium arboreum* and *Gossypium raimondii* with A_2_ and D_5_ genomes, respectively, are diploid cotton species (2n = 2× = 26) known as common parents‐like of the allotetraploid cotton (Kunbo et al. 2018). Upland cotton and Arabidopsis share a similar genome pattern, since both evolved through whole genome duplication (WGD), A and D genomes combined to give rise to AD, allotetraploid cotton (Wendel and Grover 2015). We used *G. arboreum* (Li et al. 2014), *G. raimondii* (Wang et al. 2012) and *G. hirsutum* (Li et al. 2015) due to their publicly available genome sequencing data. Moreover, *G. raimondii* is of wild origin, and many significant alleles responsible for improving cotton performance under abiotic and biotic stress conditions have been mined from their wild ancestors (Rao et al. 2006). We comprehensively described the trihelix TFs in the three cotton species, *G. hirsutum* (AD)_1_, *G. arboreum* (A_2_) and *G. raimondii* (D_5_), through comparative genome analysis. The main objectives of our study were to identify and characterize the TH TFs in cotton, to analyze their structure and conserved motifs, to undertake their functional analysis through expressing a novel cotton *TH* into the model plant *A. thaliana*, to analyze their expression patterns under drought and salt stress conditions in cotton, and finally to carry out virus‐induced gene silencing (VIGS) on the novel gene in upland cotton to understand their possible role in cotton in relation to drought and salt stresses tolerance.

## Materials and methods

### Plant materials

In this experiment, three cotton genotypes were used, *G. hirsutum* (AD_1_ accession number ZM‐30014), *G. arboreum* (A_2_; accession number ZM‐08050) and *G. raimondii* (D_5_; accession number ZM‐09213) in addition to the model plant, *Arabidopsis thaliana* (Colombia‐0 ecotype, Col‐0). The seeds of the three cotton species were germinated on a filter paper and placed in the seed germination chamber, then transferred to a hydroponic set up with Hoagland nutrient solution (Hoagland and Arnon 1950), in a climate controlled greenhouse with 16 h/8 h light–dark and temperature at 28°C day/25°C night (Zhang et al. 2017). Stress was imposed at three leaf stage, by supplementing the Hoagland nutrient solution with 15% of PEG‐6000, for drought treatment and 300 mM of NaCl solution for salt stress, untreated plants were left as controls. The root and leaf samples were collected for RNA extractions at 0, 3, 6, 12 and 24 h posttreatment. Each treatment had three replications. The samples upon collection were frozen in liquid nitrogen and stored at −80°C for RNA extraction.

### RNA isolation and real‐time quantitative polymerase chain reaction analysis

The RNA was isolated from the samples by using an RNA extraction kit, EASYspin plus plant RNA kit (Aid Lab) as per the manufacturer's instructions. Agarose gel electrophoresis was used to determine the quality and quantity of all the RNA samples extracted. RNA was then reverse transcribed into cDNA using the Prime Script RT reagent Kit (TaKaRa). Gene‐specific primers (Table S1) were used for real‐time quantitative polymerase chain reaction (RT‐qPCR) analysis. The reactions were performed using a Step one plus Real‐Time PCR System (Applied Biosystems). The PCR parameters were as follows: 94°C for 30 s, 40 cycles at 94°C for 10 s and 60°C for 30 s, and then a melting curve for 61 cycles at 65°C for 10 s was generated to check the specificity of the amplification. Relative fold expression changes were calculated using the comparative Ct value method (Heid et al. 2015).

### Identification of the trihelix (*TH*) genes

In the identification of the *TH* genes, the various proteins encoded by the *TH* genes in the three cotton species were determined. The TH proteins for *G. hirsutum* (AD)_1_ and *G. arboreum* (A_2_) were obtained from the cotton research institute website (http://mascotton.njau.edu.cn/), *G. raimondii* (D_5_), *G. max*; *Theobroma cacao* and *Oryza sativa* were downloaded from phytozome (https://phytozome), while *A. thaliana* was obtained from TAIR (http://www.arabidopsis.org/). The Hidden Markov Model (HMM) profiles of the TH functional domain PF13837 were retrieved from the Pfam database (http://pfam.xfam.org/) and quarried for the identification of the putative TH proteins with the best domain e‐value cutoffs of <1 × 10^−4^. With a cutoff e‐value of <10^−10^, the TH sequences of Arabidopsis were used as the query to perform a BLASTP search. NCBI database (http://www.ncbi) and SMART tool (http://smart.embl-heidelberg.de/) were used to analyze these potential sequences to validate the HMM and BLAST search (Ludwig‐Müller 2011). The MUSCLE program was used to perform the alignment of the full‐length protein sequences of TH with the default parameters (Edgar 2004). The maximum‐likelihood method was used to construct the phylogenetic relationship, MEGA7 was employed to calculate Bootstrap values with 1000 replications (Tamura et al. 2011).

### Chromosomal mapping, subcellular localization prediction, *cis*‐promoter, motif identification and gene structural analysis of the cotton trihelix proteins

Chromosomal position information was used for mapping the THs with Mapchart (Voorrips 2002). In addition, we carried out the subcellular localization prediction of all the TH proteins with the online tool WoLF PSORT (https://wolfpsort.hgc.jp/). We further determined various *cis*‐elements putatively binding to the various *TH* genes using an online tool PLACE database (http://www.dna.affrc.go.jp/PLACE/). In order to identify the conserved motifs of the cotton TH proteins, the online tool MEME (Bailey et al. 1997) was used with the default parameters, except for the maximum number of motifs, 10; optimum motif width 20–250. Comparing the coding sequences (CDSs) and the genome sequences of the *TH* genes, we determined the gene structure using the gene structure displayer server (http://gsds.cbi.pku.edu.cn/).

### Plant transformation and screening of *Gh_A05G2067 (GT‐2)* GT‐trihelix gene in *Arabidopsis thaliana* lines

The application of an efficient promoter is essential to develop a vector system for effective genetic transformation, and constitutive promoters are vital for the expression of the marker genes, since a promoter is a DNA sequence which do manipulates the expression of a particular gene (Xiao et al. 2010). In this research, 35S::*Gh_A05G2067 (GT‐2)* construct (cloned in pWM101) in *Agrobacterium tumefaciens* GV3101 was confirmed by the gene‐specific primer, F ‘CGGATCCATGGAGTCAAGTATGGTAG’ and R ‘GGTCGACTCACTCGCCGCCACTGCCAAT.’ *A. thaliana* plants (Col‐0) were transformed using the floral dip method as outlined by Zhang et al. (2006). The development of T_3_ was done as outlined in Lu et al. (2018). Drought and salt stress tolerance was examined among the homozygous progenies of T_3_.

### Subcellular localization of *Gh_A05G2067 (GT‐2)*

The open reading frame of *Gh_A05G2067 (GT‐2)* was amplified by PCR using the gene‐specific primers, forward ‘ACACGGGGGACTCTAGAGGATCCATGGAGTCAAGTATGGTAGA’ and reverse ‘ACTCATACTAGTCCCGGGGATCCCTCGCCGCCACTGCCAATTG.’ The PCR products were then transformed by use of pEASY‐Uni Seamless Cloning and Assembly Kit (Moradpour and Abdulah 2017) into pBI121 vector upstream of the green fluorescent protein (GFP) sequences to produce the *35S*::*Gh_A05G2067 (GT‐2)*‐GFP construct with *GT‐2‐GFP* fusion gene under the regulation of CaMV *35S* promoter. The construct was then transferred into *A. tumefaciens* strain LBA4404 and subsequently transformed into onion epidermal cells as described by Sun et al. (2007). The GFP fluorescence was observed using a Zeiss Model Axio Imager M1 Upright Fluorescent Microscope (430004‐9901‐Axio Imager.M1).

### Stress conditions and physiological measurements of *GT‐2*‐overexpressing Arabidopsis

The T_3_ seeds of *GT‐2*‐overexpressed Arabidopsis were planted on 0.5 Murashige and S koog medium (2.2 g l^−1^ basal (MS) salt mixture, PhytoTechnology Laboratories; 30 g l^−1^ sucrose; 8 g l^−1^ agarose). The plates were put at 4°C in a dark room for 3 days and then shifted to a conditioned room, at 22°C with 16 h/8 h light/dark photoperiods. After 7 days of growth, the seedlings were transplanted into pots filled with vermiculite and humus in a ratio of 1:1, and grown under normal condition for 21 days. Drought stress was initiated by total withdrawing of watering for 8 and 12 days, while salt stress was imposed by applying 250 mM of NaCl solution for the same period. Chlorophyll concentration, cell membrane stability (CMS), relative leaf water content (RLWC) and excised leaf water loss (ELWL) were determined after 8 days poststress exposure (PSE). The chlorophyll concentration was evaluated as outlined by Tetley and Thimann (1974). RLWC was evaluated as described by Barrs and Weatherley (1962). The CMS was measured by determining the electrolyte leakage from leaves following the relative conductivity method by McKay (1998). ELWL was evaluated as outlined by Clarke and McCaig (1982).

### Oxidants and antioxidants enzyme assays in *GT‐2*‐overexpressed and wild‐type Arabidopsis

We evaluated the concentration levels of catalase (CAT), peroxidase (POD) and superoxide dismutase (SOD), three major antioxidant enzymes with a profound role under abiotic stress condition in plants (del Río et al. 2018). The CAT assay was carried out as outlined by Cakmak and Marschner (1992), while SOD was determined by monitoring the inhibition of the photochemical reduction of nitro blue tetrazolium as described by Giannopolitis and Ries (1977). The POD activity was evaluated using 4‐methylcatechol as the substrate. The increase in the absorption caused by oxidation of 4‐methylcatechol by hydrogen peroxide (H_2_O_2_), was determined at 420 nm by spectrophotometer. The reaction mixture contained 100 mM disodium phosphates buffer (pH 7.0), 5 mM 4‐methylcatechol, 5 mM H_2_O_2_ and 500 μl of leaf extract summing to a volume of 3 ml. The POD determination was carried out at room temperature, and one unit of POD enzyme activity was defined as 0.001 change in the absorbance per minute (Onsa et al. 2004).

The H_2_O_2_ concentration was evaluated using a modified (NH_4_)_2_Fe(SO_4_)/xylenol orange method (Wei et al. 2002). The assay mixture with the leaf extract contained 250 μM of (NH_4_)_2_Fe(SO_4_), 100 μM sorbitol (C_4_H_14_O_6_) and 100 μM xylenol orange (C_31_H_32_N_2_O_13_S; Nile Chemicals) in 25 mM H_2_SO_4_. Based on previous research on the effects of solvents on absorbance, 1% ethanol to the assayed mixture, this was done in order to improve sensitivity of the assay. The internal standard solution was made by adding 30% H_2_O_2_ to the reagents. The H_2_O_2_ concentration was determined using absorbance at 240 nm and an extinction coefficient of 43.6 M^−1^ cm^−1^. Finally, malondialdehyde (MDA) was determined by measuring the level of lipid peroxidation as outlined by Heath and Packer (1968a, 1968b).

### RT‐qPCR analysis of the stress‐responsive genes in WT and *GT‐2*‐overexpressing Arabidopsis

RNA was isolated from 1‐month‐old *Gh_A05G2067 (GT‐2)*‐overexpressing Arabidopsis seedlings and non‐transformed wild‐type (WT) grown under drought condition. Total RNA was extracted using the RNA extraction kit, EASYspin Plus plant RNA extraction kit (Aidlab). The quality and quantity of RNA samples were determined by gel electrophoresis and NanoDrop techniques. The RNA was then reverse transcribed into cDNA using the Prime Script RT reagent Kit (TaKaRa), and used as templates in PCR analysis. Expression analysis of the stress‐responsive genes *ABF4*, *SOS1*, *CBL1* and *RD29A* on tissues obtained from *Gh_A05G2067 (GT‐2)*‐overexpressing lines and WT under drought stress condition, was carried out using the fluorescent intercalating dye FastaStart Universal SYBR‐Green Master in a detection system (Roche Diagnostics GmbH). Arabidopsis *ACTIN2* gene was used as a standard control in the RT‐qPCR reactions. Three biological replicates and three technical replicates were carried out for each cDNA sample. The stress‐responsive genes and the reference gene primers are made available (Table S2).

### Expression profiling of the *Gh_A05G2067 (GT‐2)* in various cotton tissues by RT‐qPCR

RNA was isolated from the sepal, leaf, petal, stamen, roots, stem, pistil, seed and fiber tissues of upland cotton, *G. hirsutum*, accession number ZM‐30014). The samples were collected every 3 h up to 24 h after stress exposure. The *GhACTIN7* was used as a reference gene and specific *Gh_A05G2067 (GT‐2)* genes primer forward sequence ‘ATCCAATCTTTTCTCCACT’ and reverse sequence ‘TCTTGTTTCTCAAT CACCT’ were employed for RT‐qPCR analysis.

### Preparation of inocula and inoculation of plants

The production of a Tobacco rattle virus (TRV)‐based VIGS vector suitable for *Agrobacterium*‐mediated inoculation and the production of a recombinant pTRV vector for the silencing of *Gh_A05G2067 (GT‐2)* in cotton was carried out as described by Gao and Shan (2013). A 444‐bp gene‐specific fragment from *Gh_A05G2067* was amplified by PCR using the following primers; vTH‐F (5′CGAGCTCATAGTGCGGTTTCAGTT TCG3′, *Sac*I restriction site underlined) and vTH‐R (5′CCTCGAGCCTGGTGGTTGTTTGCTTGC3′, *Xho*I restriction site underlined). Binary plasmids harboring pTRV1, and either empty pTRV2 or the recombinant pTRV2 containing *35S*::*Gh_A05G2067 (GT‐2)* were electroporated into *A. tumefaciens* strain LBA4404 and selected on plates containing kanamycin (50 μg ml^−1^) and rifampicin (50 μg ml^−1^). The bacterial culture preparation for inoculation was carried out as outlined by Gao and Shan (2013). In order to confirm the success of the gene silencing, we carried out gel electrophoresis of the RNA extracted from the leaf tissues of phytoene desaturase (PDS; positive control), WT, TRV: 00 (Control) and TRV:GT (VIGS plants) using the gene primer, F ‘CGGTTTCAGTTTCGCTTTC’ and the R ‘CCCTTTGGTCCATTGTCTT’; the TRV1 primer sequence, F ‘TTACAGGTTATTTGGGCTAG’ and R ‘CCGGGTTCAATTCCTTATC’ and the primer sequence for TRV2, F ‘TGTTTGAGGGAAAAGTAGAGAACGT’ and the R ‘TTACCG ATCAATCAAGATCAGTCGA’.

### Stress treatment and profiling of stress‐responsive genes on the VIGS cotton plants and WT

The *Gh_A05G2067‐*silenced plants (VIGS plants) leaves were observed after 15 days to ascertain the effectiveness of gene silencing. The agroinfiltrated plants were grown under normal conditions until reaching the three true leaf stage. Drought stress was imposed by complete withdrawal of water until the soil water content was below −20 kPa and then maintained 8 days; soil is known to be well watered when the soil water potential is above −30 kPa (Parent et al. 2010). Salt stress was imposed by irrigating with saline water (300 mM of NaCl). After 8 days posttreatment, chlorophyll content, CMS, ELWL and RLWC were evaluated. In addition, oxidants and antioxidant enzyme assays were carried out in the *Gh_A05G2067(GT‐2)‐*silenced plants and the WT leaves under salt and drought stress condition. Finally, expression analysis of the four abiotic stress‐responsive genes was carried out on the leaves of VIGS plants and WT cotton under drought stress condition. The stress‐responsive specific gene primers used for RT‐qPCR analysis and the conditions for the RT‐qPCR were set as described by Heid et al. (2015), *GhP5CS*‐F ‘TTGAAATAGTGGACGACGTGGC’ and *GhP5CS*‐R ‘CTCAGCGC CTAGACCAAATCG’; *GhSOD‐*F ‘CATCTCTCACGCACTCTGTC’ and *GhSOD*‐R ‘CCTTAGCCAT TTCTGTCTGTG’ and finally the *GhMYB*‐F ‘TGGGAGTAGAGGAG GAGAAGC’ and *GhMYB*‐R ‘TT GAGGTGCCTGTGGATTG.’

## Results

### Identification of trihelix proteins in cotton

We identified a total of 204 putative proteins encoded by *TH* genes among the three cotton species, with 102, 51 and 51 proteins in *G. hirsutum* (AD)_1_ *, G. raimondii* (D_5_) and *G. arboreum* (A_2_), respectively. The proteins encoded by *TH* genes were further analyzed in order to determine their physiochemical properties, unraveling diverse features. Of interest, all the cotton TH proteins had a grand average hydropathy of less than 0, which indicated that the entire TH proteins in cotton were hydrophilic in nature. Hydrophilicity is highly associated with proteins encoded by stress‐responsive genes. For instance, the majority of the late embryogenesis abundant (LEA) proteins grand average of hydropathy (GRAVY) values were found to be less than zero, an indication that the LEA proteins are hydrophilic in nature with profound roles in enhancing drought stress tolerance in cotton (Tunnacliffe and Wise 2007, Sasaki et al. 2014, Li et al. 2018). Majority of the proteins encoded by the trihelix genes in cotton species are predicted to be localized in the nucleus (43 proteins from each of the two diploid cottons, *G. arboreum* and *G. raimondii,* 83 proteins of *G. hirsutum* accounting for 81.3% of all the TH proteins). The remaining TH in *G. hirsutum*, *G. arboreum* and *G. raimondii* were found to be distributed mainly in the following four cellular structures: chloroplast, cytoplasm, endoplasmic reticulum and other extracellular structures. In *G. hirsutum* and *G. raimondii*, eight members of the trihelix proteins were found to be chloroplast proteins, six cytoplasmic proteins, three endoplasmic reticulum protein and only two were embedded within the extracellular structures, while in *G. arboreum* three, four and one proteins were found to be located in the chloroplast, cytoplasm and endoplasmic reticulum, respectively (Table S3).

### Phylogenetic analysis of cotton trihelix proteins with other plants

The cotton TH proteins were classified into six groups. Members in group 1 were mainly composed of the ARABIDOPSIS 6B‐INTERACTING PROTEIN 1‐LIKE 1 and 2 (ASIL1 and 2) TH‐type of proteins, and thus named ASIL clade. Members in group 3 were of the EMBRYO DEFECTIVE 2746 (EMB) type of the TH proteins, thus named EMB clade. Group 5 was mainly composed of the Arabidopsis SH4‐RELATED 3 (ASR3) proteins and designated as ASR3 clade. Members of group 6 are the GT clade composed of GT‐3a, GT‐3b, GTL2, PETAL LOSS (PTL) and GT‐2 (Fig. S1A). GT‐elements/motif are regulatory DNA sequences found in tandem repeats in the promoter region genes (Villain et al. 1996, Ayadi et al. 2004). Members of groups 2 and 4 contained unclassified protein of the trihelix domain, thus no clade designation was done. The classification of the cotton TH proteins was in agreement with previous publications (Kaplan‐Levy et al. 2012). In group 1, other forms of TH proteins were found to be cladded with ASIL1 and ASIL2, namely VirF‐INTERACTING PROTEINS 3 and 5 (VFP3/5) and FRIGIDA INTERACTING PROTEIN 2 (FIP2). We aligned members of each subclass of the major known clades in order to identify the most common distinctive features of the cotton TH proteins. There were high sequence similarities between the TH proteins from the three cotton species together with the sequences obtained for *T. cacao* and Arabidopsis (Fig. S1B). The high sequences similarities with the well‐characterized Arabidopsis TH proteins showed that the TH proteins derive from a common evolutionary pool.

### Genomic organization and chromosomal distribution of the cotton trihelix genes

The three cotton species exhibited different protein structures. In *G. hirsutum,* 37 TH proteins (36.3%) were found to be intronless. The remaining 63.7% (65 genes) were interrupted by introns, which ranged from 2 to 17 (Fig. S2A). The highest numbers of introns existed in *Gh_A03G0459*, *Gh_A07G0459*, *Gh_D03G1080* and *Gh_D07G0523*, while higher numbers of genes in A and D diploid cotton species were found to be intronless. For instance, in *G. arboreum* 9 (18%; Fig. S2B), and 13 (25%) in *G. raimondii* were intronless (Fig. S2C). The intronless *TH* genes in all the three cotton species were members of the ASIL clade. The highest number of intron disruption for the *TH* genes in *G. raimondii* was observed in *Gorai.003G119600* and *Gorai.001G059600*, with 17 and 18 introns, respectively. In *G. arboreum*, two genes contained the highest introns *Cotton_A_16240* and *Cotton_A_19924* with 17 each. In the two diploid cottons, the highest intron disruption was observed in the defective 2746 (emb) genes. We further analyzed the gene length (bp), exon number and mean exon lengths of all the subfamilies in the three cotton species. The *G. hirsutum TH* genes were distributed across the 26 chromosomes with only one being mapped in the scaffold region. The highest gene locus was observed in chromosome A_h_01 and its homologous D_h_01 with nine genes each closely followed by the chromosome A_h_05 and its homolog D_h_05 with eight and seven genes, respectively. The lowest gene loci were detected in chromosome A_h_03, A_h_04, A_h_13, D_h_02 and D_h_13 with a single gene locus (Fig. S3A). In the diploid cotton species, the genes were mapped in 13 and 12 chromosomes of *G. raimondii* and *G. arboreum*, respectively. In *G. arboreum,* the highest gene locus was observed in chromosome A_2_07 with eight genes (Fig. S3B), while in *G. raimondii* the highest gene loci were noted in chromosome D_5_03 and D_5_09 with nine and eight genes, respectively (Fig. S3C). A unique feature was noted on chromosome 5 across the three cotton species, in *G. raimondii*, chromosome D_5_05 contained 1 gene (*Gorai.005G079200*), A_2_05 of *G. arboreum* had zero genes, while in allotetraploid cotton, A_h_05 and its homolog D_h_05 contained eight and seven genes, respectively. This anomaly can be explained by either gene having undergone duplication or rapid gene loss among the parental lineage of the allotetraploid cotton.

### RNAseq analysis profiled under abiotic stress conditions

The majority of the genes in *G. hirsutum* were highly upregulated under salt stress condition as opposed to drought stress across the various stress levels. However, under the two stress conditions, the expressions of all the genes were clustered into three groups. In group 1, genes were highly upregulated, group 2 gathers downregulated genes while others were not expressed and in group 3, genes exhibited differential expression. Under drought stress conditions, seven genes were significantly upregulated across the leaves, roots and stems (Fig. S4A i). Similarly, under salt stress, significantly higher numbers of genes were highly upregulated (Fig. S4A ii). In the two diploid cotton species, the expression patterns of the *TH* genes were similar to those of *G. hirsutum*, the expression of the profiled genes showed upregulation, downregulation and differential expression pattern, thus were classified into three groups. In *G. arboreum* of A genome, the 15 genes of group 2 were significantly upregulated under salt stress condition (Fig. S4B i). Under drought conditions, only eight genes were highly upregulated and were clustered in group 2 (Fig. S4B ii). Finally, in *G. raimondii*, 16 genes were significantly upregulated across the three tissues under salt stress conditions (Fig. S4C i), while under drought condition, 17 genes were found to exhibit significant upregulation (Fig. S4C ii). We later carried out the RNA profiling of the various *TH* genes under normal condition in various tissues of the three cotton species to determine the tissue with the highest induction level of the *TH* genes. The expression levels of the genes in the various tissues were clustered in two groups, with one group showing significant upregulation (Fig. S4D i, iii and v). Statistical analysis of the gene expressions in the various tissues showed that more genes were inducted in pistil (79 genes), leaf (76 genes), root (72 genes) and calyx (72 genes) while stamen had the least number of upregulated genes, with only 60 genes, translating to 59% of all the genes found for *G. hirsutum* (Fig. S4D ii). Similar observations were made for the two diploid cotton species, in which pistil showed the highest level of trihelix genes expression with 42 and 40 genes in *G. arboreum* and *G. raimondii*, respectively. The lowest level of genes expression was detected in the stamen, which had 30 genes exhibiting upregulation for *G. arboreum* (Fig. S4D iv), while in *G. raimondii*, the lowest level of genes expression was observed in the petal with only 32 genes being upregulated (Fig. S4D vi).

### RT‐qPCR validation of the selected genes

We used 24, 15 and 16 genes from *G. hirsutum* (AD)_1_, *G. arboreum* (A_2_) and *G. raimondii* (D_5_), respectively, to carry out the RT‐qPCR analysis. The genes were selected based on the phylogenetic tree analysis, gene structure, subfamily classification and RNA sequence expression. In *G. hirsutum* (AD)_1_, more genes were significantly upregulated in the roots compared to the leaves (Fig. S5A), while among the two diploid cotton species, more genes showed significantly higher upregulation in the leaves as compared to the roots (Fig. S5A,B). Majority of the upregulated genes were members of the GT‐2 and ASIL1/2 subfamily. For instance, *Gh_A05G2067 (GT‐2)* was significantly upregulated among the *G. hirsutum TH* genes across the two stress levels in the root and leaf tissues. Therefore, this gene was further used in the functional analysis of the *TH* genes in cotton and Arabidopsis under salt and drought stress condition.

### Subcellular location determination, RNA isolation and RT‐qPCR analysis of the transformed gene, *Gh_A05G2067 (GT‐2)*

**Figure 1:**
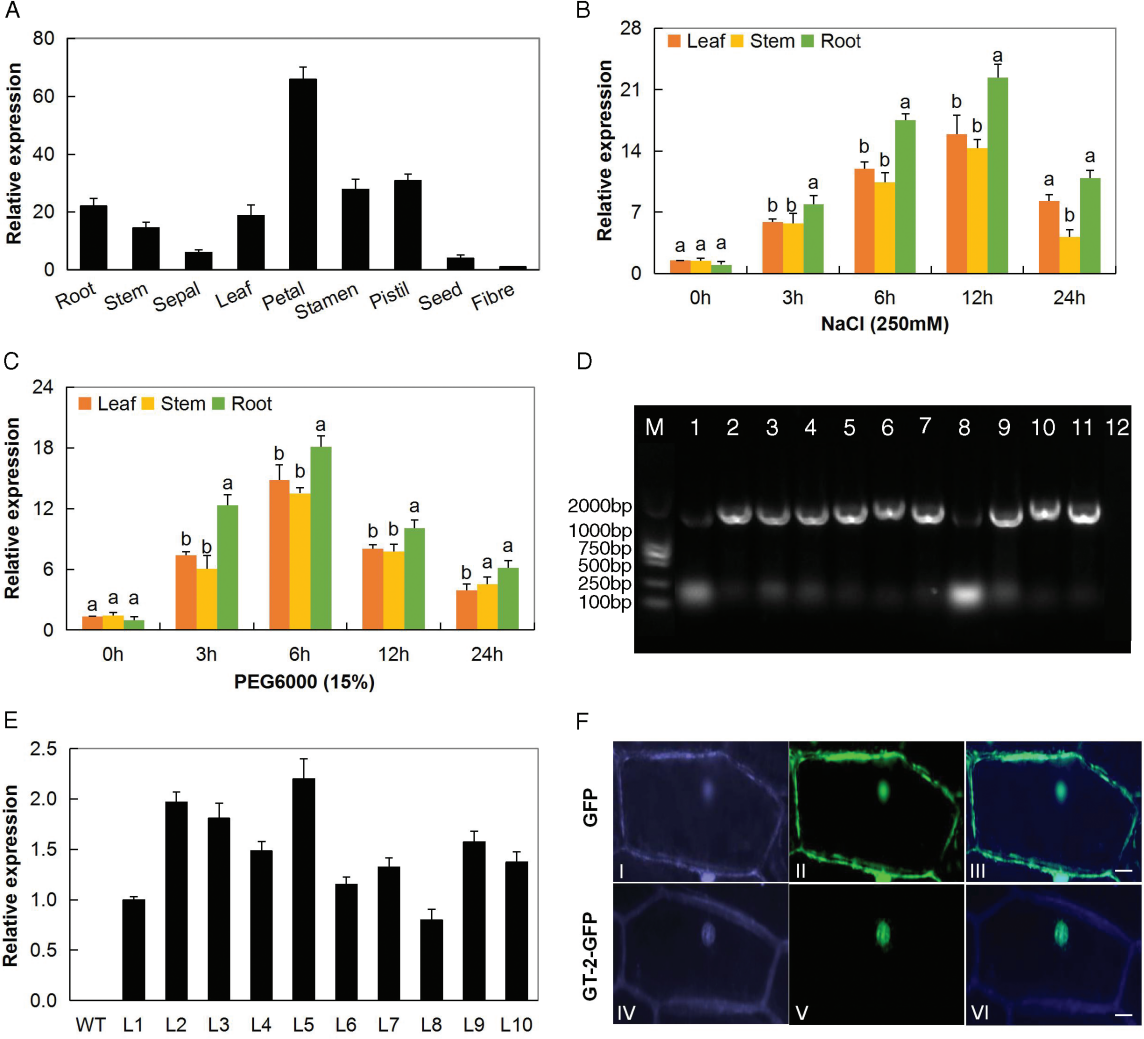
Expression analysis of *Gh_A05G2067 (GT‐2)*. (A) Relative expression of *Gh_A05G2067 (GT‐2)* gene in different parts of upland cotton *Gossypium hirsutum* under normal conditions. (B–C) Expression analysis of *Gh_A05G2067* in leaf, root and stem of (B) salt‐stressed or drought‐stressed (C) upland cotton *G. hirsutum*. (D) PCR analysis performed to check the 1737‐bp CDS integration in transformed Arabidopsis plants at T1 generation, number 1–10: *GT‐2*‐overexpressed lines, 11: positive control (plasmid containing the gene) and 12 is the negative control (WT). (E) Expression analysis of *Gh_A05G2067* in the *GT‐2*‐overexpressed Arabidopsis lines at T2 generation. (A, B, C, E) Error bars represent the sd of three biological replicates. Letters a/b indicated statistically significant differences (two‐tailed, *P* < 0.01) (F) Subcellular localization determination of Gh_A05G2067 in onion epidermal cells. Onion epidermal cells transformed with (I–III) 35S::GFP, (IV‐VI) 35S::Gh_A05G2067‐GFP. (I, IV) Bright field to display morphology, (II, V) GFP fluorescence and (III, VI) merged images. Scale bars, 10 μm.

The expression analysis showed that *Gh_A05G2067 (GT‐2)*, was highly abundant in the reproductive tissues, such as the pistil, but still high levels were detected in vegetative tissues such as the leaf, root and the stem (Fig. 1A). Expression analysis of the overexpressed gene in various tissues of cotton under drought and salt stress conditions showed that the gene was significantly upregulated in the root as opposed to the other tissues (Fig. 1B,C). Out of the 10 independent Arabidopsis overexpressed lines (Fig. 1D), L2, L3 and L5 showed the highest level of overexpression of the gene (Fig. 1E) and were thus used to carry out phenotypic and other functional evaluation under drought and salt stress conditions. The fusion of the *GT‐2* gene with GFP (under 35S promoter) indicated that the protein was located within the nucleus (Fig. 1F).

### Oxidant and antioxidant enzymes assays in *Gh_A05G2067 (GT‐2)*‐overexpressed and WTs under drought and salt stress condition

**Figure 2:**
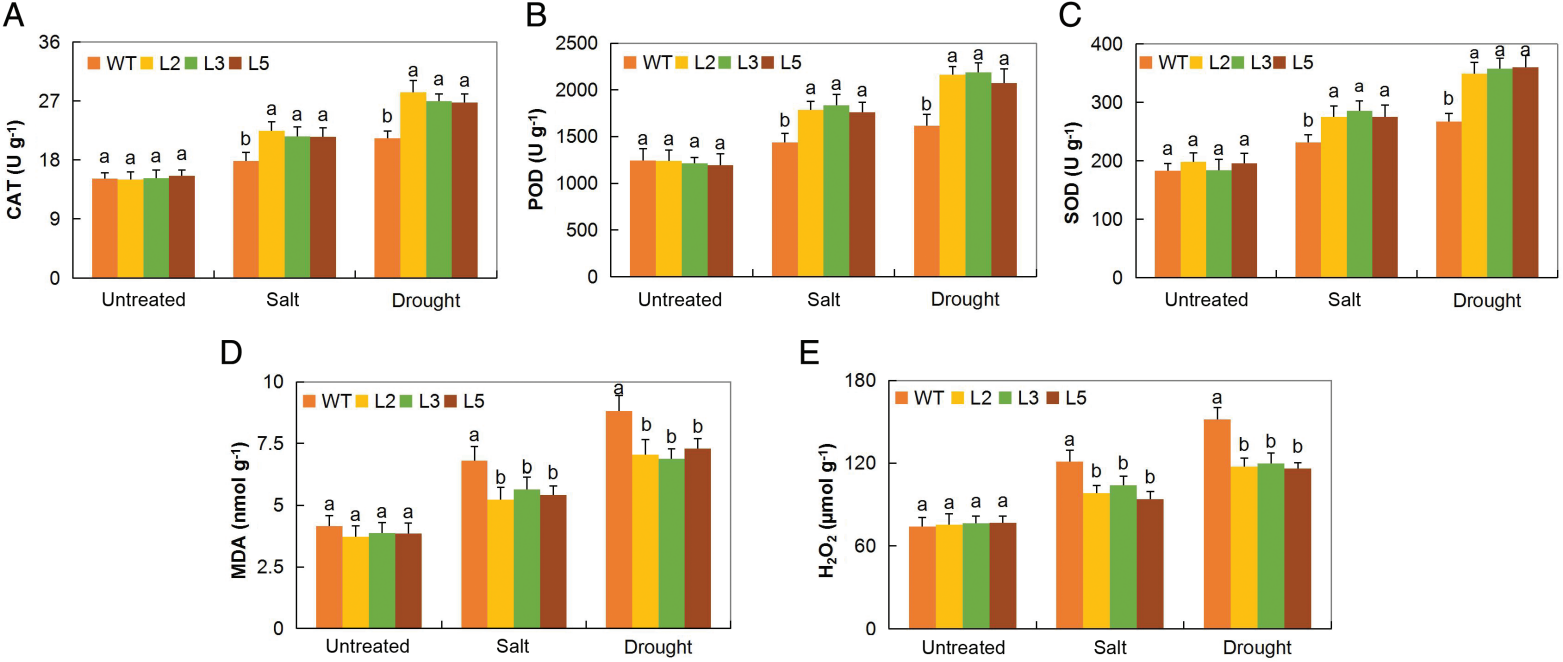
Oxidants and antioxidants contents *GT‐2*‐overexpressed and WT Arabidopsis. Quantitative determination of (A) CAT, (B) POD,(C) SOD, (D) MDA and (E) H_2_O_2_ concentrations in leaves of WT and *GT‐2*‐overexpressed lines (L2, L3 and L5) after 8 days PSE. In (B–E), each experiment was repeated three times. Bar indicates se. Different letters indicate significant differences between WT and *GT‐2*‐overexpressed lines (anova; *P* < 0.05). Untreated: normal conditions. The letters a/b indicate statistically significant differences (two‐tailed, *P* < 0.01).

The activity of CAT, SOD and POD in the leaves of the *Arabidopsis GT‐2*‐overexpressed lines was significantly higher than in the WT (Fig. 2A–C). The increased levels of antioxidant enzymes in the *GT‐2‐*overexpressed lines showed that the transgenic lines had the ability to significantly reduce the ROS to non‐toxic levels under salt and drought stress conditions. An antioxidant such as the glutathione has been found to increase highly in the stress‐tolerant maize genotype (Z7) as compared with the stress‐sensitive one (Penjalinan) after being exposed to abiotic stress conditions (Kocsy et al. 2008). Finally, we found that the levels of oxidants (H_2_O_2_ and MDA) were significantly higher in the WT but significantly lower in the *GT‐2‐*overexpressed Arabidopsis lines under drought and slat stress conditions (Fig. 2D,E).

### Physiological traits evaluation under drought and salt stress conditions

**Figure 3:**
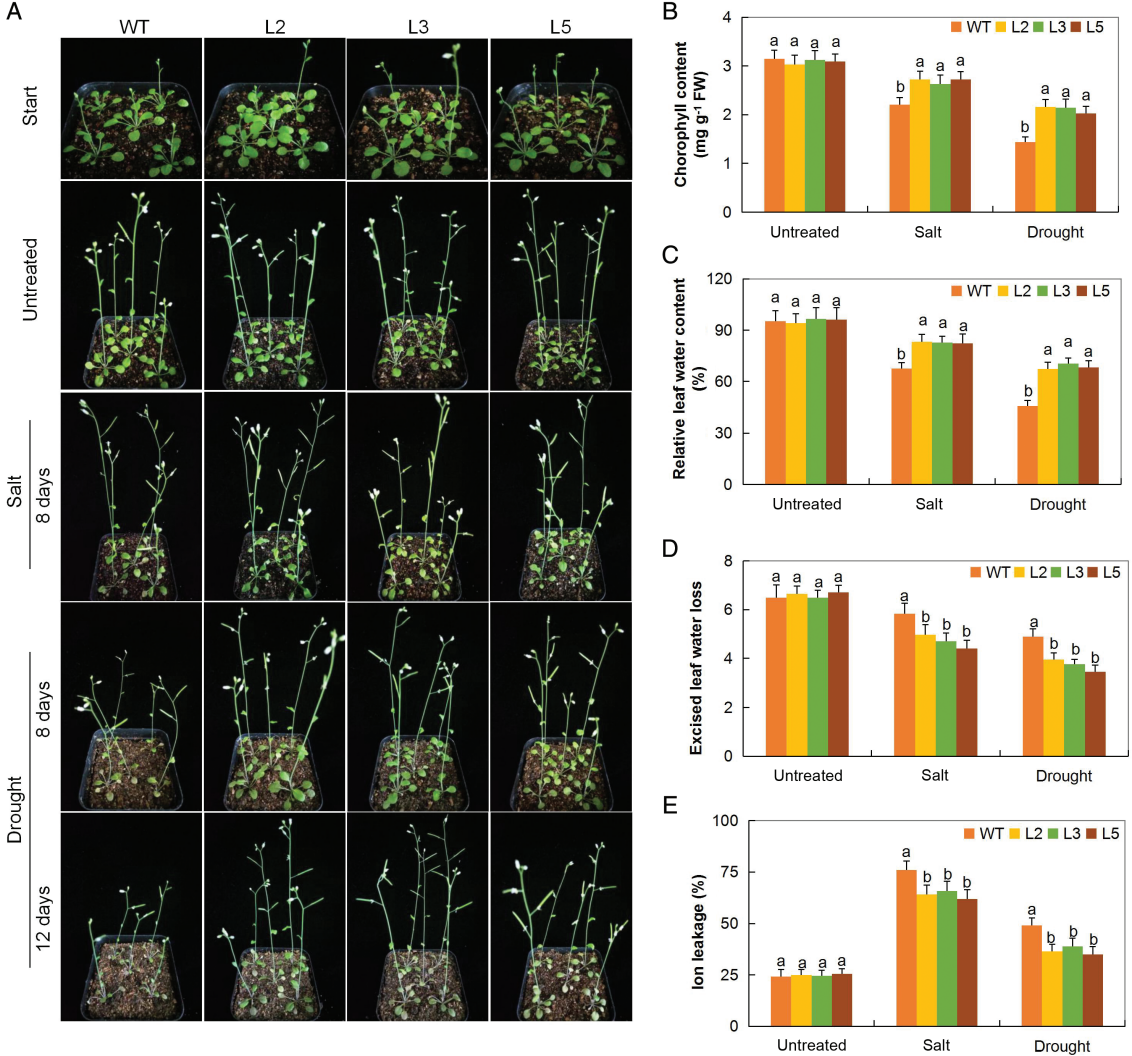
Physiological traits measurements in *GT‐2*‐overexpressed lines under salt and drought stress conditions. (A) Representative images of *GT‐2*‐overexpressed lines and WT plants before and after salt and drought treatments. (B) Quantitative determination of (C) chlorophyll content, (D) RLWC, (E) ELWL, (F) CMS as a measure of ion leakage concentration in leaves of WT and *GT‐2*‐overexpressed lines (L2, L3 and L5) 8 days PSE. In (B–E), each experiment was repeated three times. Bar indicates se. Different letters indicate significant differences between WT and *GT‐2*‐overexpressed lines (two‐tailed; *P* < 0.05). Untreated: normal conditions.

The chlorophyll content variation among the *GT‐2‐*overexpressed lines were insignificant, but it was significantly lower in WT as compared to the *GT‐2*‐overexpressed Arabidopsis lines (Fig. 3A,B), which is in agreement with previous findings showing that total chlorophyll content (expressed per unit dry weight) in wheat cultivars with varying levels of drought tolerance increased insignificantly during the first two periods of drought but decreased by 13–15% later (Nikolaeva et al. 2010). Similarly, RLWC was found to be significantly higher among the *GT‐2‐*overexpressed lines compared to the WT (Fig. 3C). In relation to ELWL, there was a significant reduction in water loss among the *GT‐2‐*overexpressed lines compared to the WT (Fig. 3D). CMS determined through ion leakage percentage was relatively low among the overexpressed plants compared to the WT (Fig. 3E).

### Transcripts investigation of stress‐responsive genes in the tissues of *GT‐2‐*overexpressed lines and WTs under drought and salt stress conditions

**Figure 4:**
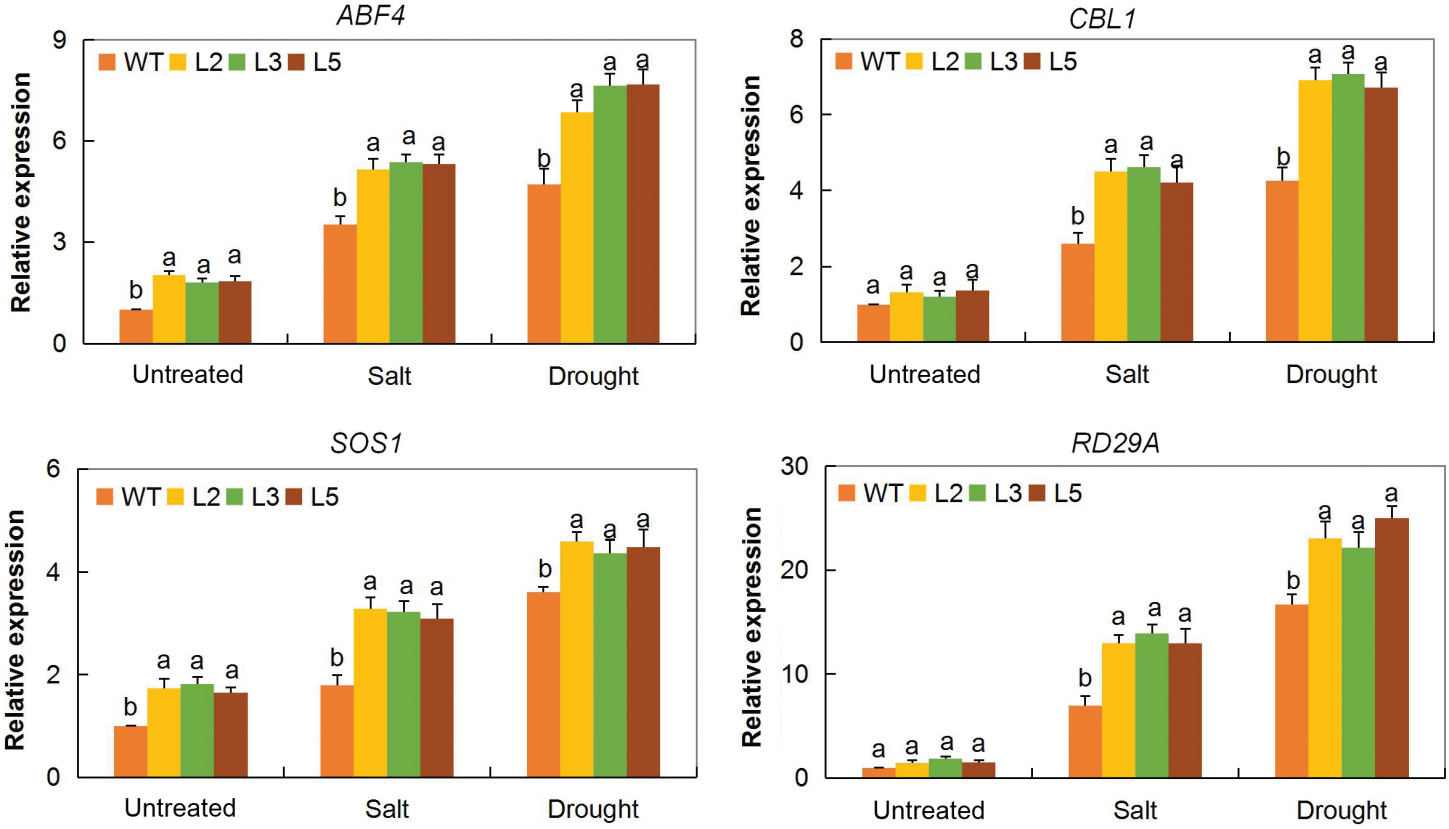
Expression levels of abiotic stress‐responsive genes (*ABF4*, *CBL1*, *SOS1* and *RD29A*) in *GT‐2*‐overexpressed lines (L2, L3 and L5) and WT Arabidopsis. *AtACTIN2* was used as the reference gene. The letters a/b indicate statistically significant differences (two‐tailed, *P* < 0.01). Error bars of the gene expression levels represent the sd of three biological replicates.

We analyzed known stress‐responsive genes in the *GT‐2‐*overexpressed Arabidopsis lines (L2, L3 and L5) to determine if *Gh_A05G2067* had a role under drought and salt stress. Four stress‐responsive genes were used, *CALCINEURIN B‐LIKE PROTEIN 1* (*CBL1*), *ABRE BINDING FACTOR 4* (*ABF4*), *DESICCATION‐RESPONSIVE RD29A* (*RD29A*) and SOS *RAS/RAC GUANINE NUCLEOTIDE EXCHANGE FACTOR 1* (*SOS1*). All genes were found to be highly upregulated in *Gh_A05G2067 (GT‐2)* overexpressed lines under the two stresses (Fig. 4). This significant upregulation showed that the *Gh_A05G2067* had a role in enhancing drought and salt stress tolerance. Overexpression of the *CBL1* gene in Arabidopsis has been found to increase their tolerance to water deficit and salinity stresses (Cheong 2003). Abscisic acid (ABA), a plant phytohormone, is the key regulator of abiotic stress, especially in water deficit resistance in plants (Kim et al. 2010). The AREB/ABF (ABA‐responsive element‐binding protein/ABA‐binding factor) plays a significant role in ABA‐dependent signaling pathways as a regulon (Yoshida et al. 2010).

### Physiological traits evaluation in the *Gh_A05G2067 (GT‐2)* *‐*silenced plants under drought and salt stress conditions

After 12 day postinoculation (dpi) in upland cotton, *G. hirsutum* seedlings infected with *Agrobacterium* carrying the *PDS* gene, the TRV–PDS construct, the albino phenotype was observed on the leaves and the stem region on the plants. Plants with the characteristic albino phenotype had a silencing efficiency of over 99% at 20 dpi due to the widespread of the albino appearance on the leaf surface, which extend even to the stem region. To further determine the efficiency of VIGS, RT‐qPCR assays were performed on leaf, stem and root tissues collected from the plants containing the TRV2:00 empty vectors and TRV: *GT‐2‐*construct. The transcript level of the target *GT‐2‐*gene decreased in the *GT‐2‐*silenced plants compared with that in the TRV2:00 empty vector‐infected plants in all tissues, though VIGS was relatively higher on the leaves compared to other tissues (Fig. S6A–C). These results collectively suggested that the target gene was successfully knocked down in the cotton plant.

**Figure 5:**
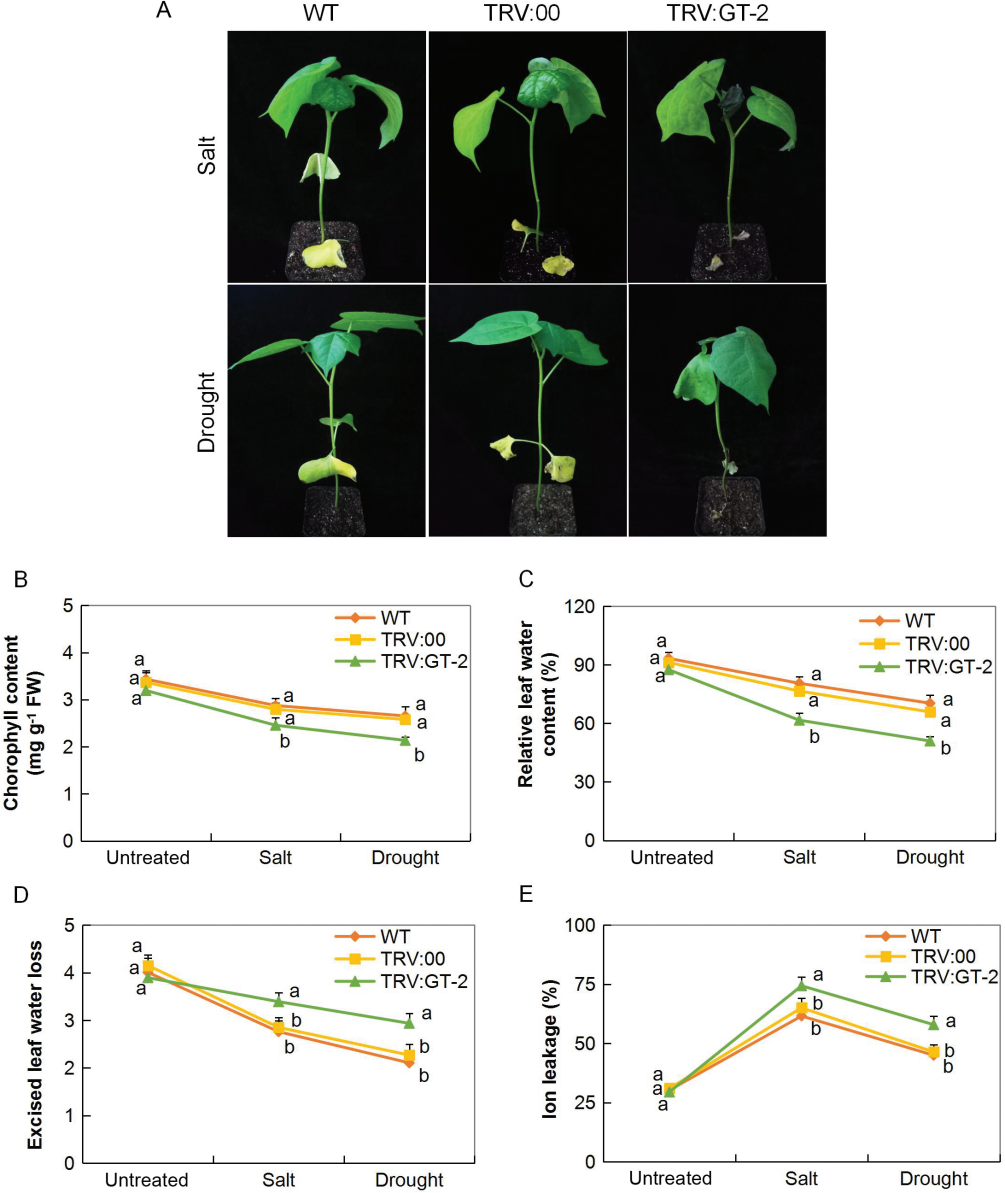
Physiological traits measurements in *Gh_A05G2067 (GT‐2)*‐VIGS cotton plants under salt and drought stress conditions. (A) Representative images of VIGS (TRV:GT‐2, silenced; TRV:00, control) and the non‐VIGS (WT) plants after 8 days of drought and salt stress treatment. (B) Quantitative determination of (C) chlorophyll content, (D) RLWC, (E) ELWL, (F) CMS as a measure of ion leakage concentration in leaves of WT, control and *GT‐2*‐silenced VIGS, evaluation done after 8 days of stress exposure. In (B–E), each experiment was repeated three times. Error bars of the physiological traits measurements represent the sd of three biological replicates. Different letters indicate significant differences between WT and G*h_A05G2067 (GT‐2)‐*VIGS plants (two‐tailed; *P* < 0.01).

In the evaluation of the various physiological traits, the *GT‐2*‐silenced plants exhibited a significant reduction in all the traits measured under drought and salt stress conditions compared to the WT and the controlled plants (Fig. 5A–E). The reduction in CMS, evidenced by a high ion leakage, showed that *GT‐2*‐silenced plants lost the ability to regulate the excess of reactive oxygen species (ROS) produced consequently to drought and salt stress conditions. Under abiotic stress condition, limitation of CO_2_ uptake due to stress‐induced stomatal closure enhances photorespiratory production of H_2_O_2_ in the peroxisome and production of superoxide and H_2_O_2_ or singlet oxygen by the over‐reduced photosynthetic electron transport chain (Choudhury et al. 2017). Excess of ROS due to stress conditions in plant cells is highly reactive and toxic to lipids, proteins and nucleic acid, which ultimately results in cellular damage and death (Rao and Chaitanya 2016).

### Oxidants and antioxidant enzyme assays under drought and salt stress conditions in *Gh_A05G2067 (GT‐2)* VIGS plants

**Figure 6:**
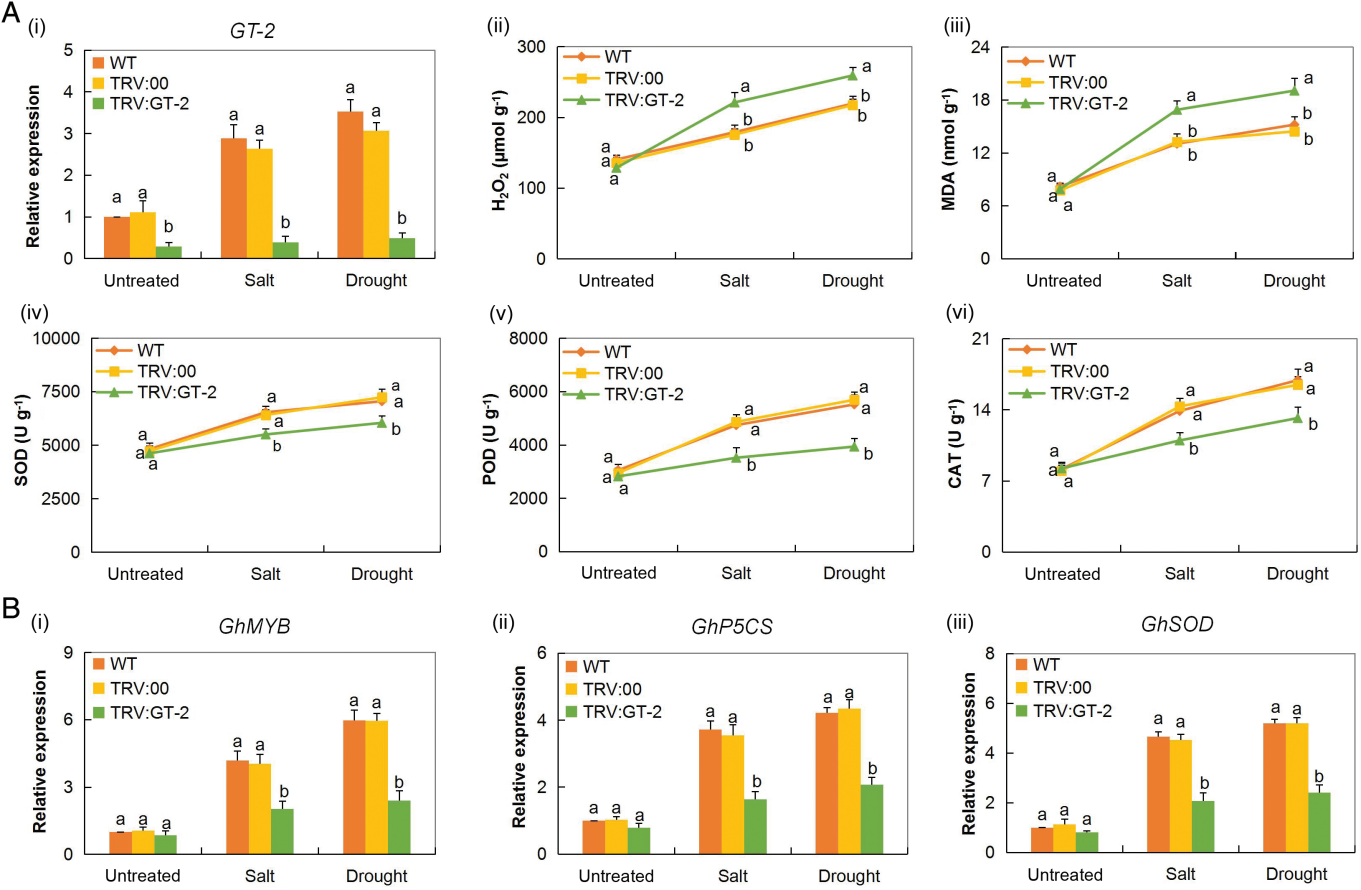
Analysis of (A) oxidant and antioxidant enzymes and (B) expression level of stress‐responsive genes in leaves of the *GT‐2*‐VIGS cotton plants under salt and drought stress conditions. (Ai) RT‐qPCR analysis confirmed that *Gh_A05G2067* is highly downregulated in TRV: *GT‐2*‐silenced cotton plant leaf. (Aii–vi) Quantitative determination of (ii) H_2_O_2_, (iii) MDA, (iv) SOD activity, (v) POD activity and (vi) CAT activity in WT, control (TRV:00) and TRV:*GT‐2* VIGS plants 8 days salt or drought treatment. In (ii–vi), each experiment was repeated three times. Bar indicates se. Different letters indicate significant differences between WT and VIGS plants (anova; *P* < 0.05). Untreated: normal conditions. (B) Relative expression of (i) *GhMYB*, (ii) *GhP5CS* and (iii) *GhSOD* stress‐responsive genes,. The relative expression level was calculated with 2^−ΔΔCT^ and normalized to the expression level of internal reference gene *GhACTIN*. The letters a/b indicate statistically significant differences (two‐tailed, *P* < 0.01). Error bars of the gene expression levels represent the sd of three biological replicates.

To determine the effect of drought and salt stresses on the VIGS plants, we determined the concentration of the oxidant and antioxidant enzymes in leaves of the VIGS plants. The antioxidants, CAT, POD and SOD were significantly reduced while the oxidants, H_2_O_2_ and MDA levels had a sharp increase in the *GT‐2*‐silenced plants but were significantly reduced in the leaves of the WT (Fig. 6A).

### Transcripts investigation of the abiotic stress‐responsive genes on the VIGS plants under drought and salt stress conditions

The expression level of cotton *SUPEROXIDE DISMUTASE* (*GhSOD*), *MYELOBLASTOSIS (GhMYB)* and *DELTA‐1‐PYRROLINE‐5‐CARBOXYLATE SYNTHETASE* (*GhP5CS*) stress‐responsive genes were downregulated under stress conditions, in the leaf tissues of the *GT‐2*‐silenced plants compared with the wild and the controlled plants (Fig. 6B). Several studies have shown that *MYB* genes have a profound effect on enhancing stress tolerance in plants. For instance, *ZmMYB30* gene a member of the *R2R3*‐*MYB* gene family has been found to be highly inducted under abiotic stress conditions in maize and its ectopic expression in transgenic Arabidopsis plants promoted salt‐stress tolerance (Mondal and Roy 2018). The downregulation of the *GhMYB* gene in the leaves of the *GT‐2‐*silenced plants indicated that the gene has a role in enhancing salt and drought stress tolerance in cotton. Moreover, *GhP5CS* and *GhSOD* genes were also downregulated in the *GT‐2‐*silenced plants.

## Discussion

The growth and development of crops are significantly affected by various abiotic stresses, which include water deficit, salt stress and extreme temperatures (Suzuki et al. 2014). Thus, breeders aim at elucidating and improving the understanding of the plant stress signaling mechanisms and initiation of adaptive‐responsive mechanisms geared toward reducing the stress effects and boosting the chances of survival (Xiong and Zhu 2001). Under the erratic weather pattern, low precipitation, extreme temperature and increased salt toxicity in arable lands, the plants have evolved multiple complex regulatory networks that detect and produce signals modulating the expression of myriad of plant TFs with diverse roles in stress tolerance (Huang et al. 2012). One of the plant TFs family highly associated with stress tolerance is the trihelix (Kaplan‐Levy et al. 2012). We carried out a genome‐wide identification of cotton trihelix genes and identified a total of 204 genes, with 102, 51 and 51 genes being distributed in *G. hirsutum* (AD)_1_ *, G. raimondii* (D_5_) and *G. arboreum* (A_2_), respectively. The number of trihelix found in the various cotton species is in agreement with previous findings, in which the members of the trihelix family have been found to be relatively low compared to other plant TFs family, such as MYBs, despite that trihelix genes are believed to have evolved from the MYBs (Nagano 2000). To date, 30, 31, 52 and 80 trihelix gene members have been identified in Arabidopsis (Kaplan‐Levy et al. 2012), *Oryza sativa* (Qin et al. 2014), *Brassica rapa* (Wang et al. 2017) and *Populus trichocarpa* (Wang et al. 2016), respectively. The low numbers of the trihelix genes in plants show that they are the most recent genes to evolve, and this could be linked to the environmental changes the plants are currently being exposed to. The number of genes detected in the diploid cotton was higher, compared to those identified in Arabidopsis, despite that Arabidopsis evolved through WGD. Similarly, the number of genes found in the two diploid cotton is similar to those obtained for *Brassica rapa*, which is considered as a mesopolyploid type of plant, but it does function as a diploid plant (Wang et al. 2011).

We further analyzed the gene length, exon number and mean exon lengths of all the *TH* subfamilies in the three cotton species. Most of the cotton *TH* genes were interrupted by introns, the highest intron interruptions were observed in *Gh_A03G0459*, *Gh_A07G0459*, *Gh_D03G1080* and *Gh_D07G0523* with 17 introns, in which all were members of the *RIBONUCLEASE J* (*RNJ*) genes. Interesting to note that the four genes also had in common two functional domains: Zn‐dependent metallo‐hydrolase RNA specificity domain (PF07521) and metallo‐β‐lactamase superfamily (PF00753). The latter is a protein domain contained in the β‐beta‐lactamases and a number of other proteins (Carfi et al. 1995). Metallo‐β‐lactamases are important enzymes because they are involved in the breakdown of antibiotics by antibiotic resistant bacteria (Brem et al. 2016). A previous report has shown that *GLYOXALASE* II (*GLYII*), belonging to the superfamily of metallo‐β‐lactamases and overexpression of *OsGLYII‐2* gene in tobacco and *Escherichia coli* enhanced tolerance to dicarbonyl and salinity stress (Ghosh et al. 2014). On the other hand, some *TH* genes were intronless, as it is the case for 9 (18%) in *G. arboreum* and 13 (25%) in *G. raimondii*. The intronless *TH* genes in all the three cotton species were members of the Arabidopsis *6B‐INTERACTING PROTEIN 1‐LIKE 1 AND 2* (*ASIL1/2*).

Physiochemical properties of all the cotton species were somehow similar, with GRAVY values of less than zero. The GRAVY values are important protein property; it does indicate the behavior of the protein type to cellular water. The GRAVY values of TH proteins in all the three cotton species ranged from −1.397 to −0.324. A very low GRAVY value implies that the protein is more soluble in water than those with a higher GRAVY index (Kyte and Doolittle 1982), and thus the cotton TH proteins are hydrophilic in nature. Numerous hydrophilic proteins have been found to enhance abiotic stress tolerance. For instance, the proteins encoded by the *LEA* genes were found to be hydrophilic, more so the dehydrin types (Magwanga et al. 2018b). The dehydrin‐like proteins have been found to be integral in plants during drought stress exposure, they protect the cells from dehydration stress (Hanin et al. 2011).

The protective role of the dehydrin proteins against cell dehydration could be due to the cryoprotective role of the dehydrin in macromolecular stabilization by binding water molecules to their hydrophilic surfaces, which reverses or prevents further denaturation of cellular proteins (Close 1996). The hydrophilic nature of the TH proteins could probably be playing the protective role within the plant cells against dehydration occasioned by exposure to water deficit condition. In addition, hydrophilic proteins have also been found to be important in reducing the amount of oxidants produced within the plant cells during stressful conditions. Abiotic stress such as drought triggers a wide variety of plant responses, including alterations in gene expression, the accumulation of metabolites such as the phytohormone ABA or osmotically active compounds, and the synthesis of specific proteins, especially hydrophilic proteins scavenging the oxygen radicals released by the plants (Huseynova et al. 2016). This shows that the TH proteins are critical in plants response to various abiotic stresses.

The entire activities within the cell are controlled by the nucleus and thus, the nucleus requires an efficient signaling mechanism in order to communicate with other cellular structures (Guilluy and Burridge 2015). The chloroplast is the main organ in which ROS is produced. The coordination between the nucleus and the chloroplast is important, as ROS can trigger signaling pathways that initiate either programmed cell death or adjustment to changed conditions (Apel and Hirt 2004). In all the three cotton species, the proteins encoded by the trihelix genes were majorly found to be nucleus localized: 83 (81.3%), 43 (84.3%) and 32 (62.7%) in *G. hirsutum* (AD)_1_, *G. arboreum* (A_2_) and *G. raimondii* (D_5_), respectively. This is in agreement with previous findings in which most trihelix proteins have been identified to be localized in the nucleus (Brewer 2004). Despite the high number of nucleus‐localized TH, others were found to be localized in the chloroplast, cytoplasm, endoplasmic reticulum and extracellular structures, which indicates other regulatory roles. The hydrophilicity and high concentration of these proteins within the nucleus explains the vital role played by these proteins in signal and initiation of various adaptive responses to various abiotic stress factors.

RNA sequence analysis and the RT‐qPCR validation showed that members of the GT‐2 subfamily were highly upregulated under drought and salt stress conditions. Gene expression profiling provides fundamental information on the possible roles of genes in plants (Livak and Schmittgen 2001). More genes were upregulated in the leaf and root tissues compared to the stem. The leaf and root tissues are the most affected organs under any form of environmental stresses (Minh‐Thu et al. 2013, Vishal and Kumar 2018). The leaf being the primary organ for photosynthesis, excessive release of ROS degrades the chlorophyll, resulting in chlorotic symptoms which affect the process of photosynthesis (Sourour et al. 2017). The increased expression levels of these genes in leaf tissues showed that they could be having a role in regulating the ROS production and thus reducing oxidative stress within the photosynthetic cells.

When plants are exposed to various environmental stresses, such as drought and salinity, the cellular activities and various biochemical pathway dynamics shift (Harrison 2012). The cellular respiration leads to excessive production of ROS, which caused massive oxidative damage which eventually leads to plant death. Among the tolerant plants, the excessive release of ROS results in mobilization of antioxidant enzymes which regulate the level of ROS within the cell (Gill and Tuteja 2010). In this research work, the *GT‐*overexpressed Arabidopsis plants were found to have higher levels of CAT, POD and SOD as opposed to the WT. The increased levels of these antioxidants under drought and salt stress conditions indicated that the transformed gene had a regulatory role in mobilizing the antioxidant enzymes to catalyze the ROS to non‐toxic levels, thus minimizing the oxidative damage. It is worth noting that ROS in plants is released as normal byproduct in various pathways including photosynthesis. It has been found that every 1–2% of oxygen taken in by plants is inevitably converted to ROS such as perhydroxyl radical, hydroxyl radicals and superoxide radical (Katekhaye and Kale 2012). Excessive accumulation of ROS results in oxidative damage to cellular and organelle membranes through peroxidation of lipids (Zhang et al. 2015). Lipid peroxidation is the most deleterious process known to occur in any living organism and occurs when the ROS is above the threshold tolerable by the cell. The extent of lipid peroxidation is measured by the level of MDA concentration (Yadav et al. 2016). The MDA and H_2_O_2_ concentrations were significantly reduced in the transformed plants as compared to the WT, which indicated that the transformed lines had a higher capacity to tolerate salt and drought stress, leading to low or normal production of ROS, with no or minimal effects within the plant.

## Author contributions

R.O.M., K.W. and F.L. designed the experiment; R.O.M., P.L., J.N.K. and X.Y. implemented and collected the data. ROM analyzed the results and prepared the manuscript. P.L., J.N.K, X.C., Y.X., X.X.W., Z.Z., X.Y., R.N., S.G.A., J.P.H., B.H.Z., F.L. and K.W. revised the manuscript. All authors reviewed and approved the final manuscript.

## Supporting information


**Fig. S1.** Phylogenetic relationship of *TH* genes in three cotton species and other plants
**Fig. S2.** Phylogenetic tree, gene structure and motif compositions of the *TH* genes in cotton.
**Fig. S3.** Chromosome mapping of the Trihelix genes.
**Fig. S4.** RNAseq data expression profiling under abiotic stress conditions.
**Fig. S5.** RT‐qPCR validation of the selected genes under abiotic stress conditions.
**Fig. S6.** Phenotype observed in the silenced plants with the TRV2:00 empty vector, WT plants and *Gh_A05G2067 (GT‐2)‐*silenced plants at 12 days post inoculation.
**Table S1**. *TH* gene specific primers for RT‐qPCR analysis.
**Table S2.** Arabidopsis stress responsive genes primer sequences for RT‐qPCR analysis.
**Table S3.** Physiological parameters and subcellular localization of the cotton trihelix proteins.
