# Supplementary material for: Genome wide identification of the trihelix transcription factors and overexpression of Gh_A05G2067 (GT‐2), a novel gene contributing to increased drought and salt stresses tolerance in cotton
Source: Physiol Plant. 2019 Feb 13;167(3):447–64. doi: 10.1111/ppl.12920 (PMC6850275; doi:10.1111/ppl.12920)
Supplement: Supplementary file 1 — Fig. S1. Phylogenetic relationship of TH genes in three cotton species and other plants Fig. S2. Phylogenetic tree, gene structure and motif compositions of the TH genes in cotton. Fig. S3. Chromosome mapping of the Trihelix genes. Fig. S4. RNAseq data expression profiling under abiotic stress conditions. Fig. S5. RT‐qPCR validation of the selected genes under abiotic stress conditions. Fig. S6. Phenotype observed in the silenced plants with the TRV2:00 empty vector, WT plants and Gh_A05G2067 (GT‐2)‐silenced plants at 12 days post inoculation. Table S1. TH gene specific primers for RT‐qPCR analysis. Table S2. Arabidopsis stress responsive genes primer sequences for RT‐qPCR analysis. Table S3. Physiological parameters and subcellular localization of the cotton trihelix proteins. [file PPL-167-447-s001.pdf]

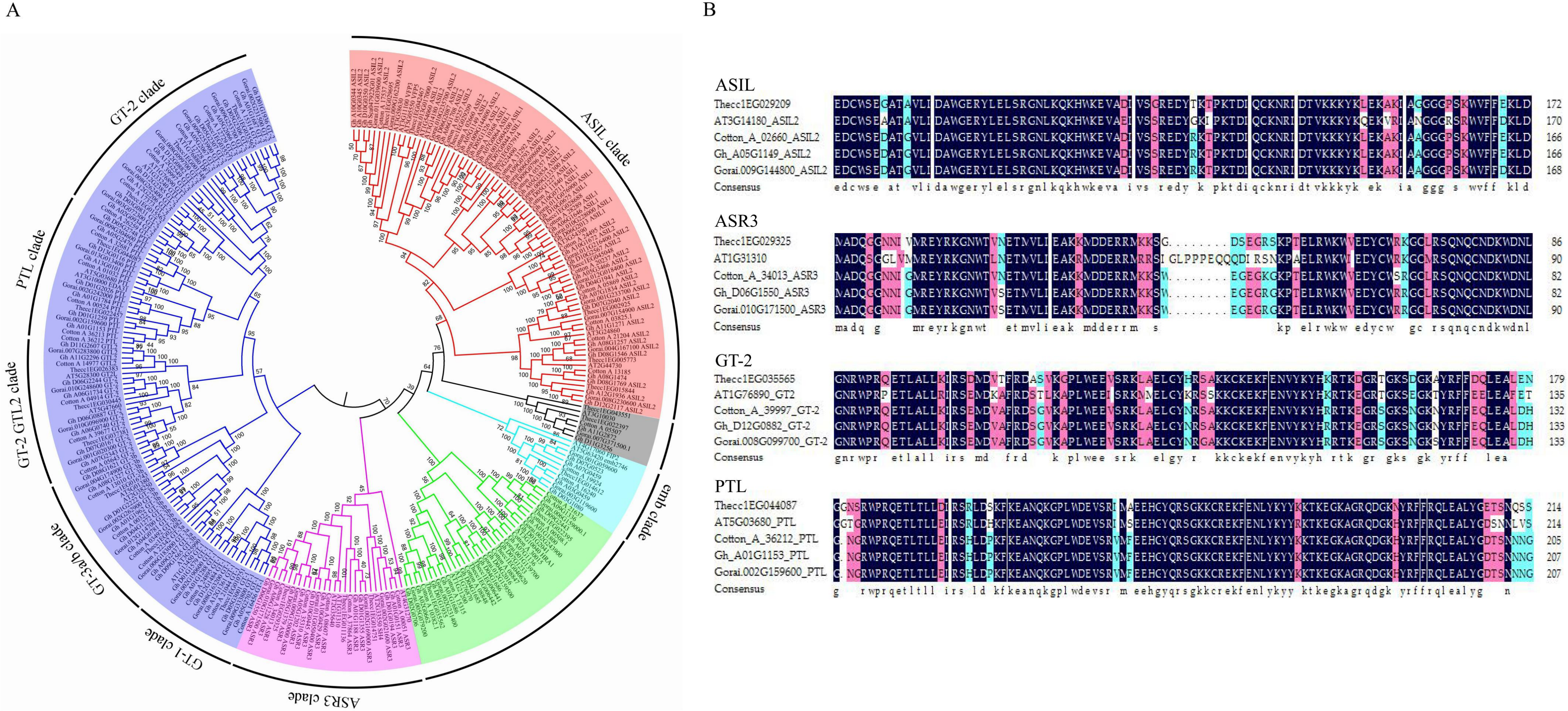


**Fig. S1:** Phylogenetic relationship of *TH* genes in three cotton species and other plants.

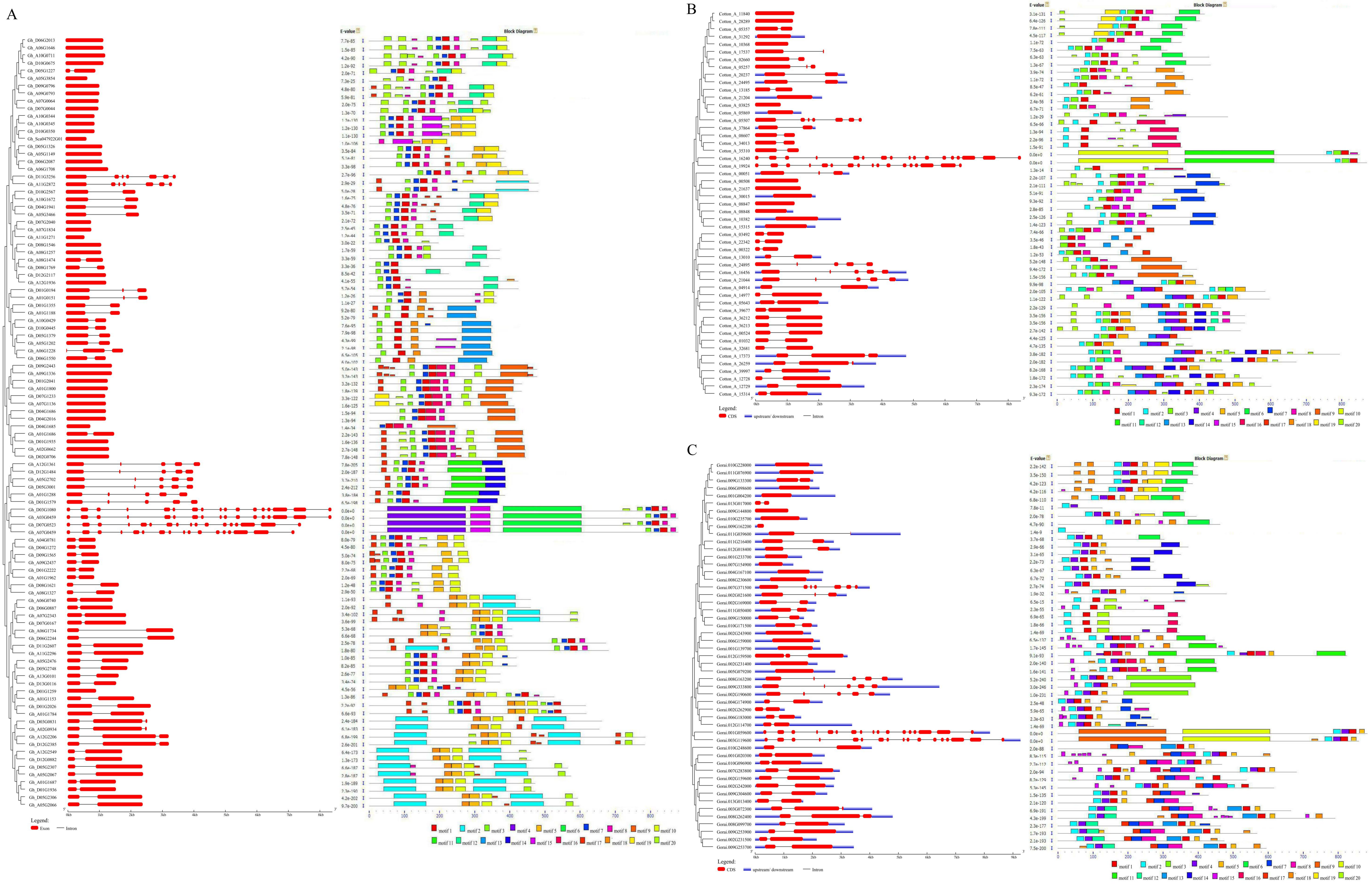


**Fig. S2:** Phylogenetic tree, gene structure and motif compositions of the *TH* genes in cotton.

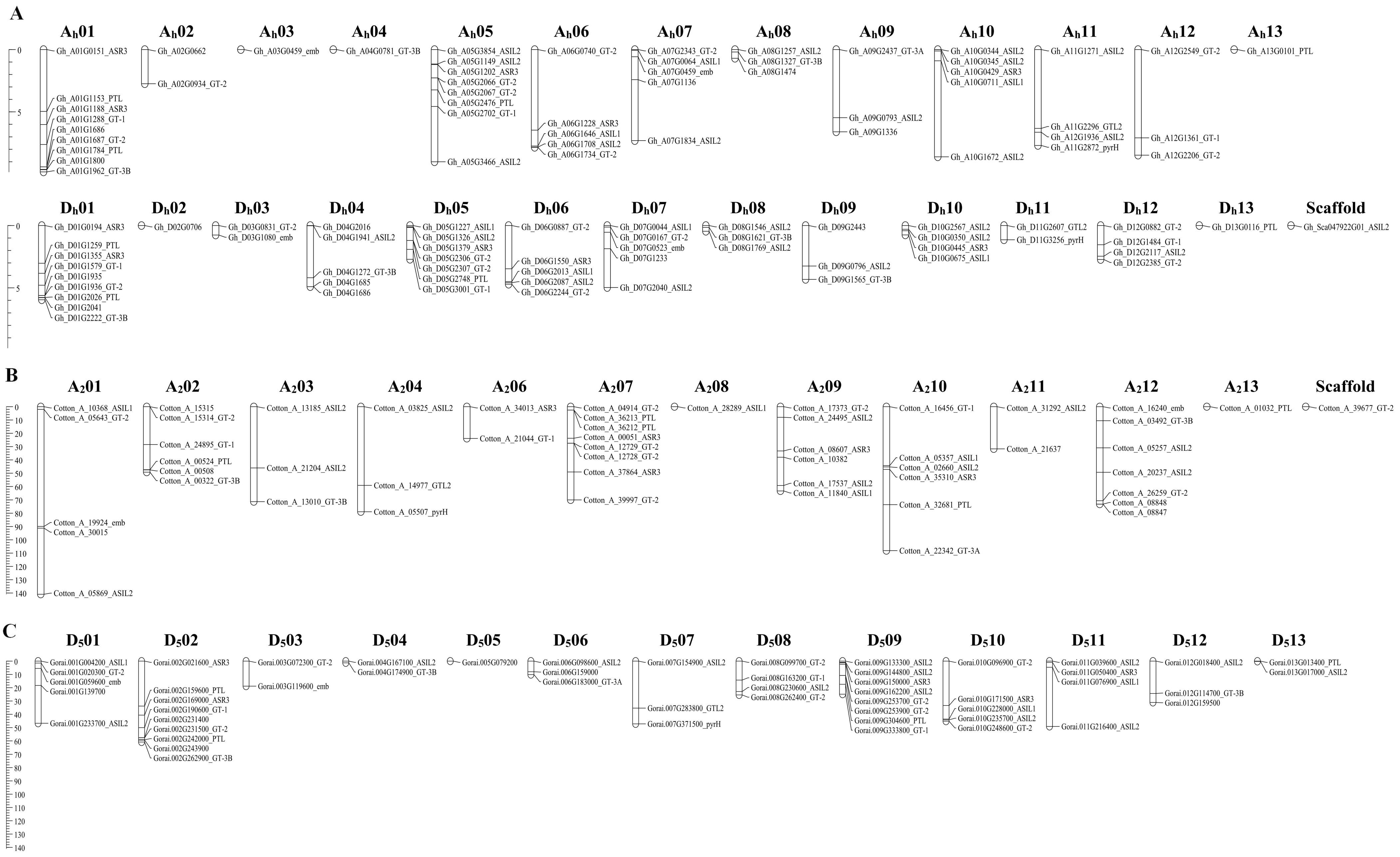


**Fig. S3:** Chromosome mapping of the Trihelix genes.

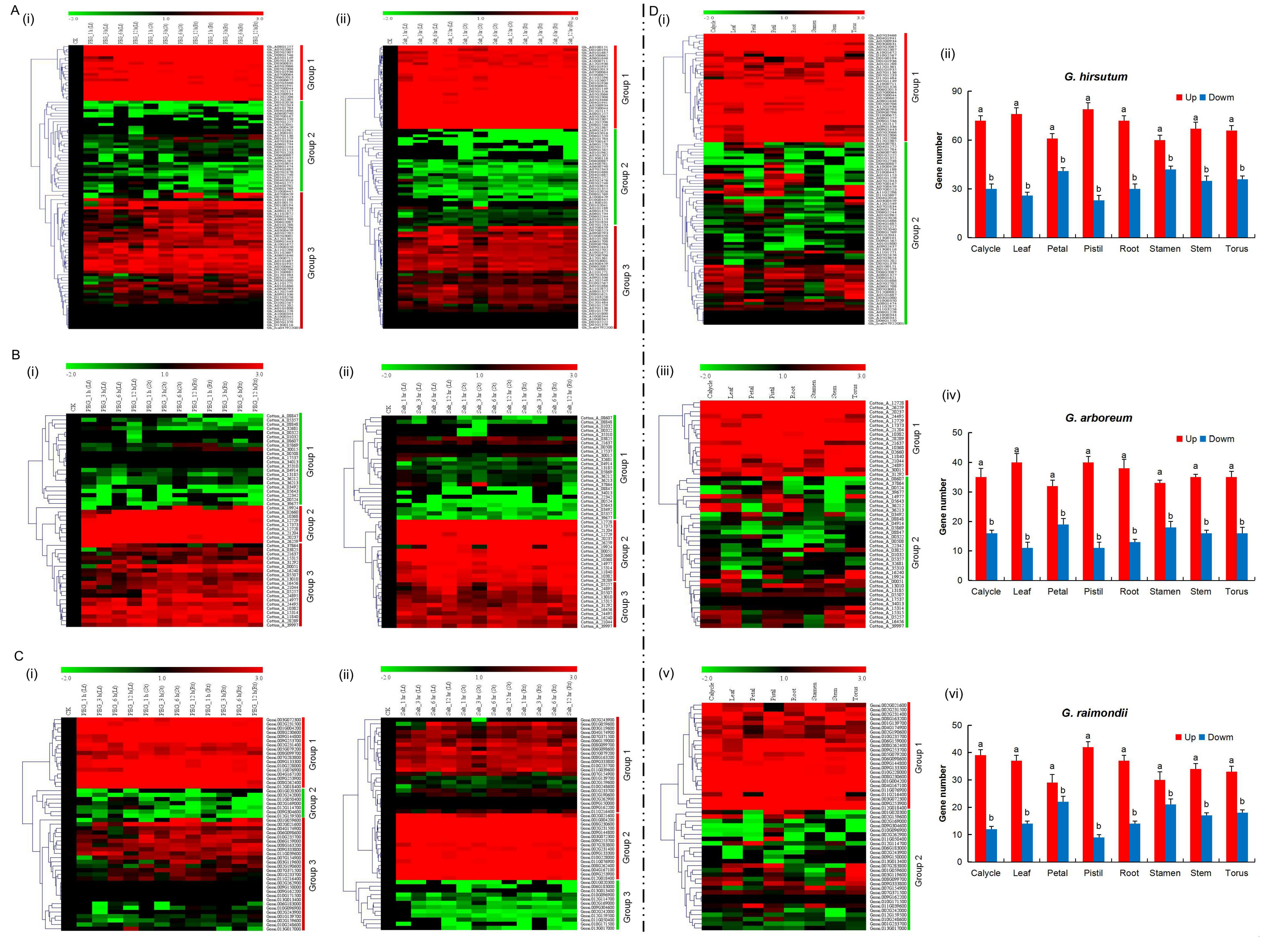


**Fig. S4:** RNAseq data expression profiling under abiotic stress conditions. The heat map was constructed using Mev.exe program. (Showed by log 2 values) in control, and in treated samples at 1, 3, 6 and 12h of salt and drought stress treatment. RNAseq expression of the trihelix genes of (A i-ii) upland cotton G. hirsutum, (B i-ii) the A-genome G. arboretum, (C i-ii) D-genome G. raimondii under drought and salt stress conditions (ANOVA; $P < 0.05$). (D i-vi) Trihelix expression in various cotton tissues under normal condition. Red: up-regulated, green-down-regulated and black-no significant difference in expression levels compared to control. Letter a/b indicated statistically significant differences (two-tailed, $P < 0.01$). Error bars of the represent the standard deviation of three biological replicates.

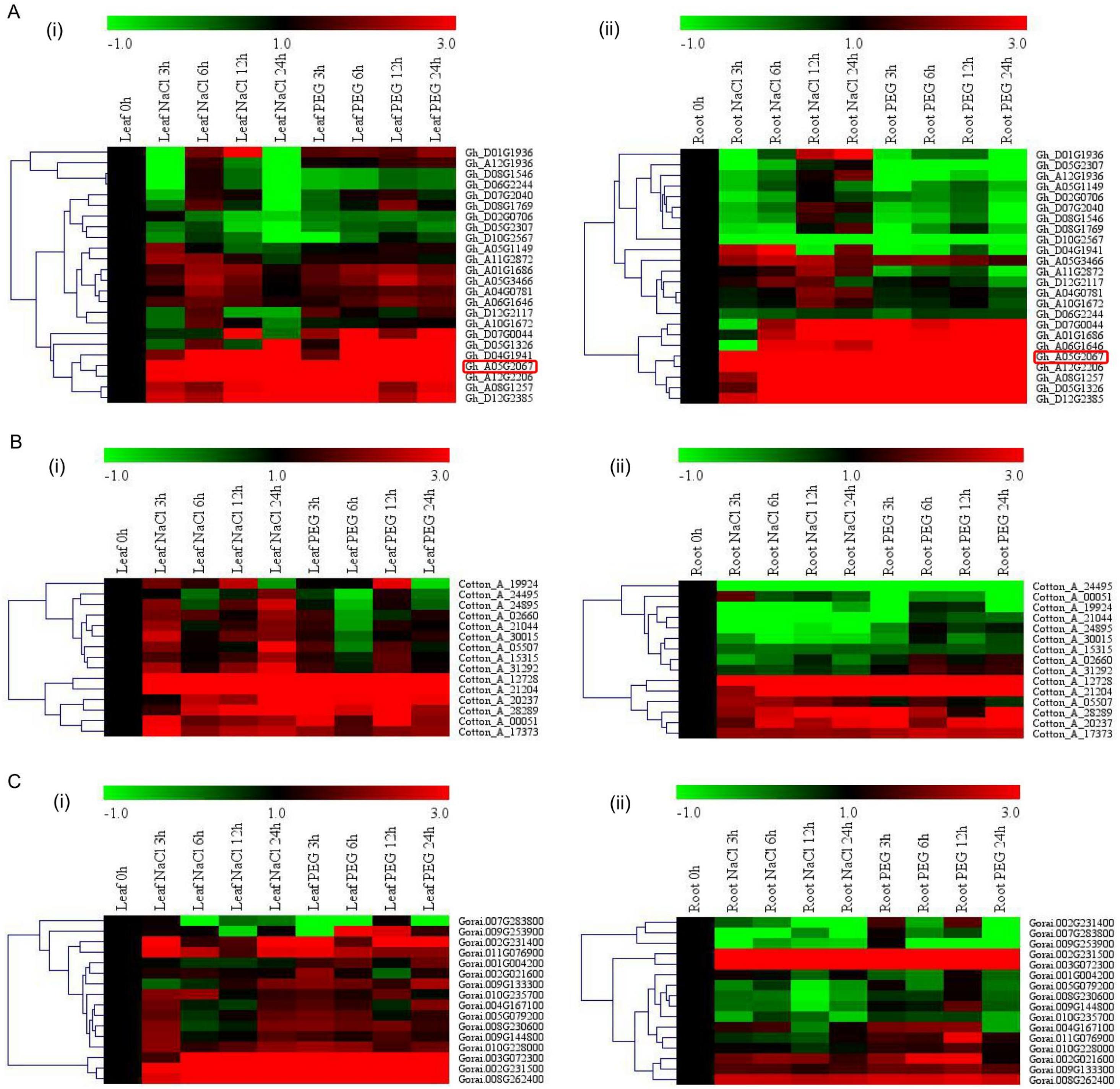


**Fig. S5:** RT-qPCR validation of the selected genes under abiotic stress conditions. The heat map was realized using Mev.exe program (Showed by log 2 values) in control, and in treated samples at 0, 3, 6, 12 and 24h of salt and drought stress treatment. Expression analysis of the trihelix genes of (A) upland cotton G. hirsutum, (B) G. arboreum and (C) G. raimondii in (i) leaf or (ii) root upon drought or salt stress Red: up-regulated, green: down-regulated and black-no significant difference in expression levels compared to control (ANOVA; $P < 0.05$).

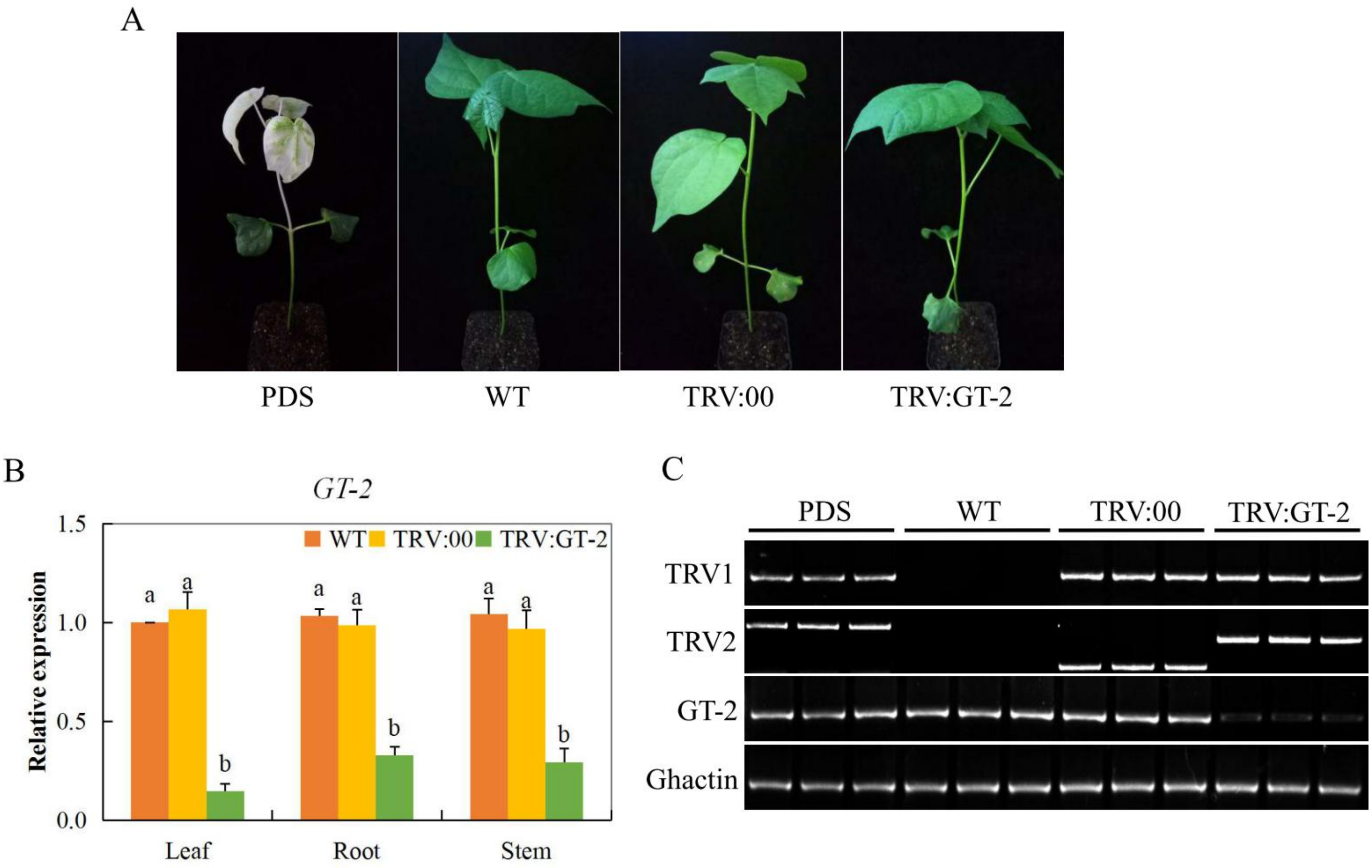


**Fig. S6:** Phenotype observed in the silenced plants with the TRV2:00 empty vector, wild-type plants and *Gh_A05G2067 (GT-2)*-silenced plants at 12 days post inoculation.

**Table S1**: *TH* gene specific primers for RT-qPCR analysis

| A. Gene specific primers for tetraploid cotton *G. hirsutum* (24 primers) | | |
|---|---|---|
| Gene | F(5'-3') | R(5'-3') |
| Gh_A01G1686 | ATCATCACCACCCTAACCC | ACATTTCCCTATTTCCATC |
| Gh_A04G0781 | AGAGGATAATGATGGAGAG | AGAAGTGCAGTAATGAGAG |
| Gh_A05G1149 | CCATCTCCGTCCTCCCTATC | CTCATGTAACTGCGGCTCTT |
| Gh_A05G2067 | ATCCAATCTTTTCTCCACT | TCTTGTTTCTCAATCACCT |
| Gh_A05G3466 | AAATGGGTGCTTAGAAATA | AGTCATCAAAGGAGGGTTA |
| Gh_A06G1646 | AGAAAGGTTATGATGGAAAG | TGAAACTGCTGGTGCTGGAA |
| Gh_A08G1257 | CGAGGGAGATAGAGACGAT | TTAGGACTGAGGTAAGGGC |
| Gh_A10G1672 | ATAACCAAGAAAACCCATC | TCAGCCTATCATTACTCCC |
| Gh_A11G2872 | CTCTGATTCTGCTTTCCG | CTCTTCTTGGTCCCTCCT |
| Gh_A12G1936 | GCTGAGGAAGCGTTACCG | ATAGAATCAAGCAGGGGA |
| Gh_A12G2206 | GGAGTTCTTTGAGGGTTTA | TTGCCATCTCTTGTCTTTT |
| Gh_D01G1936 | TGTTCTTCCAAATCCAATC | TCTTCTTCCTCATCATCGT |
| Gh_D02G0706 | ACCACCCTAACCCTCGCCA | TTTTCCCCCTCTCCCCCTT |
| Gh_D04G1941 | AAGAAGACTCCTCCCCTA | TTCACTCCATTCATCACG |
| Gh_D05G1326 | TCCCTATCACCTTCATCA | TGTAACTGCGACTCTTCC |
| Gh_D05G2307 | AATCTTTTCTCCACTTCCAC | CTCTTGTTTCTCAATCACCT |
| Gh_D06G2244 | GTTCCACATCACGGGCTCTA | TTCCCATTTCTCTTTACACT |
| Gh_D07G0044 | GCTTCCAATACGCACCTG | ACCCAATCTTCACCCCTC |
| Gh_D07G2040 | TAAGAAACCCCAACCACT | TTACGGCGACCTCCTCCC |
| Gh_D08G1546 | AAACCCAAATCCTAACCC | CCTCTTCCCTGATGCTGC |
| Gh_D08G1769 | AGGCTTACAAGGAAAAATGG | AAGATACAGCGGAAGACACG |
| Gh_D10G2567 | GAAAACCCATCTCTCCTCTC | TTCAGCCTATCATTACTCCC |
| Gh_D12G2117 | CTCCTCCTCTTCCCCAAC | AGCGAACCATTTCTCCTT |
| Gh_D12G2385 | GGGCGTCAAGATGGTAAGAG | ACGGCGACAGTAGCAGGAAT |
| B. Gene specific primers for diploid cotton *G. raimondii* (16 primers) | | |
| Gene | F(5'-3') | R(5'-3') |
| Gorai.001G004200 | CCAATACGCACCTGACCATA | TCCTCACCCCTCAAACTCCT |
| Gorai.002G021600 | ACTTCGAACAAGACGACGGT | GCTGCTGCTCTGAAATAGGG |
| Gorai.002G231400 | ATCATCACCACCCTAACCC | AACTGAACATTTCCCTATT |
| Gorai.002G231500 | TTCTACTTCTTCGTCGACT | TCACCTCTTTCATTAACCT |
| Gorai.003G072300 | TATGGATGGTATTTTTCG | TACTTGTGGACGTTCTCG |
| Gorai.004G167100 | TTCTTGGGTCCACTTTAA | TTCGTCATCTGGGCTATC |
| Gorai.005G079200 | TGATTCCTGGTGGGGGTT | GGCGAGGGTTAGGGTGGT |
| Gorai.007G283800 | TTGATGGAGTGCCAGACC | GAGAGAGGGAGAAGGAGG |
| Gorai.008G230600 | GTGCCCCAAGGTATTCATCA | CACCAGCATTCTTTCTCTCA |

| | | |
|---|---|---|
| Gorai.008G262400 | ATCTCTGCTGGTATGTCC | TTTTTGTTGCTTTCTTTG |
| Gorai.009G133300 | GAGAGACACCCAAAAAAG | TTCAGTCCAGTCCGTAAG |
| Gorai.009G144800 | AGTCTACCGCCACCTATG | ATTTCCTTCCTTTCCCTC |
| Gorai.009G253900 | TCATCCAATCTTTTCTCC | TTCCATTTCCTCTTCTTC |
| Gorai.010G228000 | TGATGAAAACGGTGATAA | ACTGCTGGTGCTGGAACT |
| Gorai.010G235700 | CACTTCCGCCGCCTCAGA | CTCCCCACGCGTCGATCA |
| Gorai.011G076900 | ATCTGGCGAGGATTCTTC | ATTTCCAACTCCCTGTGA |
| C. Gene specific primers for diploid cotton *G. arboreum* (15 primers) | | |
| Gene | F(5'-3') | R(5'-3') |
| Cotton_A_00051 | TCAAGGAATGGGAGAGTA | GTCGAGGATGTCGTAAAC |
| Cotton_A_02660 | AGTCTACCGCCACCTACG | ATTTCCTTCCTTTCCCTC |
| Cotton_A_05507 | CTCTGATTCTGCTTTCCGC | TCTCTTCTTGGTCCCTCCT |
| Cotton_A_12728 | TCATCCAATCTTTTCTCC | CCTTCCATTTTCTCTTCT |
| Cotton_A_15315 | ATCATCACCACCCTAACC | AACATTTCCCTATTTCCA |
| Cotton_A_17373 | ATCTCTGCTGGTATGTCC | GTTTTTTGTTGCTTTCTT |
| Cotton_A_19924 | AAGAAATGGAGCCACAAA | ATAACCCAAGGCAACGCA |
| Cotton_A_20237 | CTCCCCTAAAAAACACCCT | CAGCTCCTTCACTCCATTC |
| Cotton_A_21044 | GAAACCCCGTTCCATAGA | GGTGAGGTGGCAAATCCC |
| Cotton_A_21204 | ACCGAGGTGATATTGGAG | GGGCATTCTTTTGGGCTT |
| Cotton_A_24495 | AAACCCATCTCTCCTCTC | CAGCCTATCATTACTCCC |
| Cotton_A_24895 | CCACCAATGGGGGTTTAC | CTCGTTTCTTCGGGGCTT |
| Cotton_A_28289 | CGATGAAAACGGTGATAAA | GAAACTGCTGGTGCTGGAA |
| Cotton_A_30015 | AAGGTCATCAATGGTGGG | TCAAAAGCGTCTGTCTGC |
| Cotton_A_31292 | TTGAAAGGACTGACACTC | CCATCTTCTTGAAAAAAA |

**Table S2:** Arabidopsis stress responsive genes primer sequences for RT-qPCR analysis

| Gene | Forward sequence | Reverse sequence |
|---|---|---|
| *ABF4* | AACAACTTAGGAGGTGGTGGTCAT | TGTAGCAGCTGGCGCAGAAGTCAT |
| *SOS1* | TCGTTTCAGCCAAATCAGAAAGT | TTTGCCTTGTGCTGCTTTCC |
| *CBL1* | GAAATGAAACTGGCTGATGAAACCATAGAG | CTCGTGGCAATCTACTCGGTCTTAAACC |
| *RD29A* | TGAAAGGAGGAGGAGGAATGGTTGG | ACAAAACACACATAAACATCCAA AGT |
| *Atactin2* | TTGTGCTGGATTCTGGTGATGG | CCGCTCTG CTGTTGTGGTG |
| *Actin7* | ATCCTCCGTCTTGACCTTG | TGTCCGTCAGGCAACTCAT |

**Table S3:** Physiological parameters and subcellular localization of the cotton trihelix proteins.

A. Physiochemical properties of *G. hirsutum* proteins encoding the *TH* genes

| Gene ID | Gene | Description | Protein Length (aa) | Molecular Weight (kDa) | Charge | Isoelectric Point | Grand Average of Hydropathy | Transcript Length (bp) | CDS Length (bp) | CDS GC Content (%) | Exon Number | Mean Exon Length (bp) | Mean Intron Length (bp) | Chromosome | Start | End | Strand | Length (bp) | sub cellular localization |
|---|---|---|---|---|---|---|---|---|---|---|---|---|---|---|---|---|---|---|---|
| Gh_A06G1646 | ASIL1 | Trihelix transcription factor ASIL1 | 399 | 46.022 | -31 | 4.375 | -1.29 | 1200 | 1200 | 43.2 | 1 | 1200 | No intron | A06 | 101475475 | 101476674 | + | 1200 | E.R. |
| Gh_A07G0064 | ASIL1 | Trihelix transcription factor ASIL1 | 349 | 41.083 | -36 | 4.25 | -1.244 | 1050 | 1050 | 43 | 1 | 1050 | No intron | A07 | 780897 | 781946 | - | 1050 | nucl |
| Gh_A10G0711 | ASIL1 | Trihelix transcription factor ASIL1 | 419 | 48.042 | -53.5 | 4.085 | -1.246 | 1260 | 1260 | 44 | 1 | 1260 | No intron | A10 | 12125910 | 12127169 | + | 1260 | nucl |
| Gh_D05G1227 | ASIL1 | Trihelix transcription factor ASIL1 | 274 | 31.493 | -12 | 4.686 | -1.127 | 825 | 825 | 42.2 | 2 | 412.5 | 123 | D05 | 10574915 | 10575862 | + | 948 | nucl |
| Gh_D06G2013 | ASIL1 | Trihelix transcription factor ASIL1 | 399 | 45.992 | -31 | 4.369 | -1.29 | 1200 | 1200 | 43.2 | 1 | 1200 | No intron | D06 | 61841203 | 61842402 | + | 1200 | nucl |
| Gh_D07G0044 | ASIL1 | Trihelix transcription factor ASIL1 | 347 | 40.873 | -33.5 | 4.271 | -1.242 | 1044 | 1044 | 43.3 | 1 | 1044 | No intron | D07 | 489563 | 490606 | + | 1044 | nucl |
| Gh_D10G0675 | ASIL1 | Trihelix transcription factor ASIL1 | 401 | 45.8 | -29.5 | 4.371 | -1.13 | 1206 | 1206 | 43.9 | 1 | 1206 | No intron | D10 | 7579566 | 7580771 | - | 1206 | nucl |
| Gh_A05G1149 | ASIL2 | Trihelix transcription factor ASIL2 | 383 | 41.951 | 12 | 9.967 | -0.858 | 1152 | 1152 | 50.1 | 1 | 1152 | No intron | A05 | 11637659 | 11638810 | + | 1152 | E.R. |
| Gh_A05G3466 | ASIL2 | Trihelix transcription factor ASIL2 | 353 | 39.165 | 18 | 10.269 | -0.904 | 1062 | 1062 | 44.9 | 2 | 531 | 1259 | A05 | 90071926 | 90074246 | - | 2321 | plas |
| Gh_A05G3854 | ASIL2 | Trihelix transcription factor ASIL2 | 230 | 26.807 | -8 | 4.944 | -1.382 | 693 | 693 | 42 | 1 | 693 | No intron | A05 | 31206 | 31898 | + | 693 | nucl |
| Gh_A06G1708 | ASIL2 | Trihelix transcription factor ASIL2 | 450 | 49.928 | 12 | 9.654 | -0.836 | 1353 | 1353 | 45.3 | 1 | 1353 | No intron | A06 | 102322668 | 102324020 | - | 1353 | E.R. |
| Gh_A07G1834 | ASIL2 | Trihelix transcription factor ASIL2 | 269 | 31.03 | -3 | 5.764 | -0.999 | 810 | 810 | 49.4 | 1 | 810 | No intron | A07 | 73268403 | 73269212 | + | 810 | plas |
| Gh_A08G1257 | ASIL2 | Trihelix transcription factor ASIL2 | 374 | 41.727 | 13 | 9.858 | -0.954 | 1125 | 1125 | 51.6 | 1 | 1125 | No intron | A08 | 83967785 | 83968909 | + | 1125 | plas |
| Gh_A09G0793 | ASIL2 | Trihelix transcription factor ASIL2 | 357 | 41.462 | -11 | 4.868 | -1.231 | 1074 | 1074 | 41.2 | 1 | 1074 | No intron | A09 | 54750420 | 54751493 | - | 1074 | mito |
| Gh_A10G0344 | ASIL2 | Trihelix transcription factor ASIL2 | 306 | 34.078 | 6.5 | 9.503 | -0.765 | 921 | 921 | 49.3 | 1 | 921 | No intron | A10 | 3146458 | 3147378 | + | 921 | E.R. |
| Gh_A10G0345 | ASIL2 | Trihelix transcription factor ASIL2 | 306 | 34.078 | 6.5 | 9.503 | -0.765 | 921 | 921 | 49.3 | 1 | 921 | No intron | A10 | 3150366 | 3151286 | + | 921 | E.R. |
| Gh_A10G1672 | ASIL2 | Trihelix transcription factor ASIL2 | 371 | 40.918 | 14.5 | 10.035 | -0.881 | 1116 | 1116 | 46.8 | 2 | 558 | 1181 | A10 | 89153426 | 89155722 | + | 2297 | plas |
| Gh_A11G1271 | ASIL2 | Trihelix transcription factor ASIL2 | 197 | 22.74 | 1 | 6.796 | -0.927 | 594 | 594 | 49.7 | 1 | 594 | No intron | A11 | 15771395 | 15771988 | + | 594 | E.R. |
| Gh_A12G1936 | ASIL2 | Trihelix transcription factor ASIL2 | 425 | 47.7 | 9.5 | 8.726 | -0.804 | 1278 | 1278 | 43.3 | 1 | 1278 | No intron | A12 | 82117942 | 82119219 | + | 1278 | E.R. |

| | | | | | | | | | | | | | | | | | | | |
|---|---|---|---|---|---|---|---|---|---|---|---|---|---|---|---|---|---|---|---|
| Gh_D04G1941 | ASIL2 | Trihelix transcription factor ASIL2 | 349 | 38.755 | 15.5 | 10.184 | -0.89 | 1050 | 1050 | 45 | 2 | 525 | 1206 | D04 | 135530 | 137785 | - | 2256 | plas |
| Gh_D05G1326 | ASIL2 | Trihelix transcription factor ASIL2 | 388 | 42.51 | 12 | 9.967 | -0.886 | 1167 | 1167 | 49.7 | 1 | 1167 | No intron | D05 | 11657056 | 11658222 | + | 1167 | E.R. |
| Gh_D06G2087 | ASIL2 | Trihelix transcription factor ASIL2 | 390 | 42.909 | 6 | 8.955 | -0.925 | 1173 | 1173 | 46.8 | 1 | 1173 | No intron | D06 | 62773362 | 62774534 | - | 1173 | E.R. |
| Gh_D07G2040 | ASIL2 | Trihelix transcription factor ASIL2 | 267 | 30.934 | 0 | 6.536 | -1.025 | 804 | 804 | 49.5 | 1 | 804 | No intron | D07 | 50230782 | 50231585 | + | 804 | plas |
| Gh_D08G1546 | ASIL2 | Trihelix transcription factor ASIL2 | 374 | 41.708 | 13 | 9.858 | -0.972 | 1125 | 1125 | 51.6 | 1 | 1125 | No intron | D08 | 49497777 | 49498901 | + | 1125 | plas |
| Gh_D08G1769 | ASIL2 | Trihelix transcription factor ASIL2 | 227 | 25.914 | 9.5 | 9.715 | -0.522 | 684 | 684 | 47.5 | 2 | 342 | 546 | D08 | 54116520 | 54117749 | + | 1230 | nucl |
| Gh_D09G0796 | ASIL2 | Trihelix transcription factor ASIL2 | 357 | 41.704 | -11.5 | 4.877 | -1.22 | 1074 | 1074 | 41.1 | 1 | 1074 | No intron | D09 | 32529509 | 32530582 | - | 1074 | mito |
| Gh_D10G0350 | ASIL2 | Trihelix transcription factor ASIL2 | 306 | 34.079 | 6.5 | 9.503 | -0.763 | 921 | 921 | 49.4 | 1 | 921 | No intron | D10 | 3010828 | 3011748 | + | 921 | E.R. |
| Gh_D10G2567 | ASIL2 | Trihelix transcription factor ASIL2 | 372 | 40.897 | 15.5 | 10.102 | -0.869 | 1119 | 1119 | 47.6 | 2 | 559.5 | 1090 | D10 | 18541 | 20749 | + | 2209 | plas |
| Gh_D12G2117 | ASIL2 | Trihelix transcription factor ASIL2 | 424 | 47.583 | 7.5 | 8.347 | -0.808 | 1275 | 1275 | 43.5 | 1 | 1275 | No intron | D12 | 54214379 | 54215653 | + | 1275 | E.R. |
| Gh_Sca047922G01 | ASIL2 | Trihelix transcription factor ASIL2 | 221 | 24.507 | 7 | 10.023 | -0.756 | 666 | 666 | 49.5 | 1 | 666 | No intron | scaffold47922 | 184 | 849 | + | 666 | nucl |
| Gh_A01G0151 | ASR3 | Trihelix transcription factor ASR3 | 363 | 39.595 | -4 | 5.382 | -0.664 | 1092 | 1092 | 54.6 | 3 | 364 | 747.5 | A01 | 1406572 | 1409158 | + | 2587 | nucl |
| Gh_A01G1188 | ASR3 | Trihelix transcription factor ASR3 | 306 | 35.285 | 9.5 | 8.616 | -1.103 | 921 | 921 | 47.7 | 2 | 460.5 | 788 | A01 | 61519540 | 61521248 | - | 1709 | plas |
| Gh_A05G1202 | ASR3 | Trihelix transcription factor ASR3 | 347 | 38.824 | 8 | 9.211 | -0.797 | 1044 | 1044 | 46.4 | 2 | 522 | 340 | A05 | 12101735 | 12103118 | + | 1384 | mito |
| Gh_A06G1228 | ASR3 | Trihelix transcription factor ASR3 | 353 | 39.239 | 4 | 8.294 | -0.704 | 1062 | 1062 | 45.9 | 3 | 354 | 370 | A06 | 88683932 | 88685733 | - | 1802 | nucl |
| Gh_A10G0429 | ASR3 | Trihelix transcription factor ASR3 | 350 | 39.366 | 5.5 | 8.307 | -0.954 | 1053 | 1053 | 44.3 | 2 | 526.5 | 213 | A10 | 4328638 | 4329903 | - | 1266 | plas |
| Gh_D01G0194 | ASR3 | Trihelix transcription factor ASR3 | 363 | 39.691 | -4.5 | 5.24 | -0.699 | 1092 | 1092 | 54.8 | 3 | 364 | 732 | D01 | 1612007 | 1614562 | + | 2556 | nucl |
| Gh_D01G1355 | ASR3 | Trihelix transcription factor ASR3 | 307 | 35.378 | 10.5 | 8.878 | -1.099 | 924 | 924 | 47.8 | 2 | 462 | 783 | D01 | 39910861 | 39912567 | + | 1707 | plas |
| Gh_D05G1379 | ASR3 | Trihelix transcription factor ASR3 | 349 | 39.02 | 7.5 | 9.206 | -0.768 | 1050 | 1050 | 46.3 | 2 | 525 | 343 | D05 | 12112913 | 12114305 | + | 1393 | mito |
| Gh_D06G1550 | ASR3 | Trihelix transcription factor ASR3 | 338 | 37.825 | 5 | 8.576 | -0.808 | 1017 | 1017 | 46 | 2 | 508.5 | 236 | D06 | 51612518 | 51613770 | - | 1253 | nucl |
| Gh_D10G0445 | ASR3 | Trihelix transcription factor ASR3 | 350 | 39.414 | 7.5 | 8.996 | -0.968 | 1053 | 1053 | 44.4 | 2 | 526.5 | 209 | D10 | 4203561 | 4204822 | - | 1262 | plas |
| Gh_A11G2296 | At5g28300 | Trihelix transcription factor GTL2 | 682 | 77.181 | 0.5 | 6.547 | -1.005 | 2049 | 2049 | 42.3 | 2 | 1024.5 | 373 | A11 | 79024090 | 79026511 | + | 2422 | plas |
| Gh_D11G2607 | At5g28300 | Trihelix transcription factor GTL2 | 674 | 76.27 | 2.5 | 6.668 | -0.982 | 2025 | 2025 | 42.4 | 2 | 1012.5 | 378 | D11 | 54194001 | 54196403 | + | 2403 | plas |
| Gh_A01G1288 | GT-1 | Trihelix transcription factor GT-1 | 388 | 44.413 | 7.5 | 7.797 | -0.645 | 1167 | 1167 | 45 | 5 | 233.4 | 666.3 | A01 | 77535452 | 77539283 | - | 3832 | E.R. |

| | | | | | | | | | | | | | | | | | | | |
|---|---|---|---|---|---|---|---|---|---|---|---|---|---|---|---|---|---|---|---|
| Gh_A05G2702 | GT-1 | Trihelix transcription factor GT-1 | 391 | 44.71 | 2.5 | 6.738 | -0.938 | 1176 | 1176 | 46.7 | 5 | 235.2 | 711 | A05 | 45651955 | 45655974 | - | 4020 | plas |
| Gh_A12G1361 | GT-1 | Trihelix transcription factor GT-1 | 382 | 43.73 | 1.5 | 6.66 | -0.784 | 1149 | 1149 | 45.8 | 5 | 229.8 | 772.3 | A12 | 70907830 | 70912067 | + | 4238 | plas |
| Gh_D01G1579 | GT-1 | Trihelix transcription factor GT-1 | 368 | 42.346 | 2.5 | 6.844 | -0.714 | 1107 | 1107 | 44.4 | 5 | 221.4 | 759.8 | D01 | 49445363 | 49449508 | + | 4146 | E.R. |
| Gh_D05G3001 | GT-1 | Trihelix transcription factor GT-1 | 391 | 44.768 | 2.5 | 6.738 | -0.945 | 1176 | 1176 | 46.9 | 5 | 235.2 | 709.8 | D05 | 37612036 | 37616050 | - | 4015 | plas |
| Gh_D12G1484 | GT-1 | Trihelix transcription factor GT-1 | 390 | 44.878 | 4.5 | 7.077 | NA | 1173 | 1173 | 44.8 | 5 | 234.6 | 711.3 | D12 | 44985376 | 44989393 | + | 4018 | plas |
| Gh_A01G1687 | GT-2 | Trihelix transcription factor GT-2 | 475 | 54.134 | 13.5 | 9.434 | -1.005 | 1428 | 1428 | 41.8 | 2 | 714 | 96 | A01 | 95494317 | 95495840 | + | 1524 | E.R. |
| Gh_A02G0934 | GT-2 | Trihelix transcription factor GT-2 | 661 | 72.974 | 10 | 8.545 | -0.843 | 1986 | 1986 | 47.9 | 3 | 662 | 264 | A02 | 38131247 | 38133760 | + | 2514 | plas |
| Gh_A05G2066 | GT-2 | Trihelix transcription factor GT-2 | 601 | 66.411 | 2 | 6.802 | -0.963 | 1806 | 1806 | 48.8 | 2 | 903 | 569 | A05 | 22771529 | 22773903 | + | 2375 | plas |
| Gh_A05G2067 | GT-2 | Trihelix transcription factor GT-2 | 578 | 64.874 | -6.5 | 5.62 | -0.984 | 1737 | 1737 | 46.5 | 2 | 868.5 | 651 | A05 | 22777151 | 22779538 | - | 2388 | plas |
| Gh_A06G0740 | GT-2 | Trihelix transcription factor GT-2 | 458 | 51.889 | 9 | 7.948 | -0.804 | 1377 | 1377 | 41.8 | 2 | 688.5 | 71 | A06 | 24069889 | 24071336 | + | 1448 | E.R. |
| Gh_A06G1734 | GT-2 | Trihelix transcription factor GT-2 | 409 | 46.136 | -3 | 6.118 | -0.856 | 1230 | 1230 | 45.8 | 2 | 615 | 2132 | A06 | 102527881 | 102531242 | - | 3362 | nucl |
| Gh_A07G2343 | GT-2 | Trihelix transcription factor GT-2 | 593 | 67.172 | 7 | 6.929 | -0.914 | 1782 | 1782 | 40.5 | 2 | 891 | 87 | A07 | 188737 | 190605 | - | 1869 | plas |
| Gh_A12G2206 | GT-2 | Trihelix transcription factor GT-2 | 790 | 85.875 | -8.5 | 5.763 | -0.791 | 2373 | 2373 | 49.4 | 3 | 791 | 418.5 | A12 | 84784729 | 84787938 | - | 3210 | plas |
| Gh_A12G2549 | GT-2 | Trihelix transcription factor GT-2 | 464 | 53.406 | -3 | 5.996 | -1.028 | 1395 | 1395 | 41.9 | 2 | 697.5 | 329 | A12 | 44829 | 46552 | - | 1724 | nucl |
| Gh_D01G1936 | GT-2 | Trihelix transcription factor GT-2 | 475 | 53.951 | 11 | 8.885 | -1.024 | 1428 | 1428 | 42.4 | 2 | 714 | 98 | D01 | 57667441 | 57668966 | + | 1526 | plas |
| Gh_D03G0831 | GT-2 | Trihelix transcription factor GT-2 | 666 | 73.263 | 9.5 | 8.31 | -0.843 | 2001 | 2001 | 47.8 | 3 | 667 | 264.5 | D03 | 28483032 | 28485561 | - | 2530 | plas |
| Gh_D05G2306 | GT-2 | Trihelix transcription factor GT-2 | 596 | 65.914 | 5 | 7.36 | -0.963 | 1791 | 1791 | 48.6 | 2 | 895.5 | 570 | D05 | 22486770 | 22489130 | + | 2361 | plas |
| Gh_D05G2307 | GT-2 | Trihelix transcription factor GT-2 | 570 | 64.037 | -2.5 | 6.226 | -1.027 | 1713 | 1713 | 46.5 | 2 | 856.5 | 653 | D05 | 22496250 | 22498615 | - | 2366 | plas |
| Gh_D06G0887 | GT-2 | Trihelix transcription factor GT-2 | 458 | 51.979 | 11 | 8.122 | -0.793 | 1377 | 1377 | 42 | 2 | 688.5 | 70 | D06 | 16959718 | 16961164 | + | 1447 | E.R. |
| Gh_D06G2244 | GT-2 | Trihelix transcription factor GT-2 | 409 | 46.185 | -0.5 | 6.457 | -0.862 | 1230 | 1230 | 45.9 | 2 | 615 | 2173 | D06 | 63965726 | 63969128 | + | 3403 | nucl |
| Gh_D07G0167 | GT-2 | Trihelix transcription factor GT-2 | 593 | 66.72 | 11.5 | 7.358 | -0.87 | 1782 | 1782 | 40.5 | 2 | 891 | 87 | D07 | 1751729 | 1753597 | + | 1869 | plas |
| Gh_D12G0882 | GT-2 | Trihelix transcription factor GT-2 | 465 | 53.65 | -2 | 6.18 | -1.026 | 1398 | 1398 | 41.8 | 2 | 699 | 324 | D12 | 29613066 | 29614787 | + | 1722 | nucl |
| Gh_D12G2385 | GT-2 | Trihelix transcription factor GT-2 | 789 | 85.539 | -9.5 | 5.666 | -0.79 | 2370 | 2370 | 49.9 | 3 | 790 | 423.5 | D12 | 56887658 | 56890874 | - | 3217 | plas |
| Gh_A09G2437 | GT-3A | Trihelix transcription factor GT-3a | 288 | 34.652 | 4 | 7.665 | -1.336 | 867 | 867 | 45.3 | 2 | 433.5 | 153 | A09 | 124045 | 125064 | - | 1020 | vacu |

| | | | | | | | | | | | | | | | | | | | | |
|---|---|---|---|---|---|---|---|---|---|---|---|---|---|---|---|---|---|---|---|---|
| Gh_A01G1962 | GT-3B | Trihelix transcription factor GT-3b | 259 | 31.619 | 10 | 9.143 | -1.3 | 780 | 780 | 39.6 | 2 | 390 | 86 | A01 | 99587512 | 99588377 | - | 866 | E.R. |
| Gh_A04G0781 | GT-3B | Trihelix transcription factor GT-3b | 273 | 32.575 | 6.5 | 8.621 | -1.203 | 822 | 822 | 46.5 | 2 | 411 | 92 | A04 | 52520361 | 52521274 | + | 914 | E.R. |
| Gh_A08G1327 | GT-3B | Trihelix transcription factor GT-3b | 261 | 31.329 | 2 | 8.205 | -1.397 | 786 | 786 | 48.6 | 2 | 393 | 724 | A08 | 85923159 | 85924668 | - | 1510 | cyto |
| Gh_D01G2222 | GT-3B | Trihelix transcription factor GT-3b | 259 | 31.496 | 8 | 8.701 | -1.246 | 780 | 780 | 40.5 | 2 | 390 | 86 | D01 | 61117248 | 61118113 | - | 866 | E.R. |
| Gh_D04G1272 | GT-3B | Trihelix transcription factor GT-3b | 273 | 32.658 | 6 | 8.335 | -1.252 | 822 | 822 | 45.7 | 2 | 411 | 92 | D04 | 41839174 | 41840087 | + | 914 | E.R. |
| Gh_D08G1621 | GT-3B | Trihelix transcription factor GT-3b | 261 | 31.214 | 1 | 6.967 | -1.348 | 786 | 786 | 48 | 2 | 393 | 862 | D08 | 50958751 | 50960398 | - | 1648 | cyto |
| Gh_D09G1565 | GT-3B | Trihelix transcription factor GT-3b | 285 | 34.249 | 4 | 7.665 | -1.304 | 858 | 858 | 45.5 | 2 | 429 | 153 | D09 | 43234234 | 43235244 | + | 1011 | vacu |
| Gh_A01G1686 | NA | NA | 444 | 51.051 | 2 | 6.649 | -1.016 | 1335 | 1335 | 42.1 | 2 | 667.5 | 178 | A01 | 95476593 | 95478105 | + | 1513 | plas |
| Gh_A01G1800 | NA | NA | 437 | 50.139 | -7.5 | 5.726 | -1.169 | 1314 | 1314 | 38 | 1 | 1314 | No intron | A01 | 97777217 | 97778530 | + | 1314 | plas |
| Gh_A02G0662 | NA | NA | 449 | 51.603 | -4.5 | 6.155 | -1.14 | 1350 | 1350 | 42.7 | 1 | 1350 | No intron | A02 | 10737903 | 10739252 | - | 1350 | plas |
| Gh_A07G1136 | NA | NA | 416 | 47.333 | -8 | 5.24 | -1.001 | 1251 | 1251 | 43.8 | 1 | 1251 | No intron | A07 | 24370060 | 24371310 | - | 1251 | nucl |
| Gh_A08G1474 | NA | NA | 340 | 37.92 | 5 | 7.986 | -0.575 | 1023 | 1023 | 45 | 2 | 511.5 | 157 | A08 | 90940825 | 90942004 | + | 1180 | nucl |
| Gh_A09G1336 | NA | NA | 482 | 54.468 | -2 | 6.364 | -1.075 | 1449 | 1449 | 45.7 | 1 | 1449 | No intron | A09 | 66055644 | 66057092 | - | 1449 | plas |
| Gh_D01G1935 | NA | NA | 442 | 50.724 | 3 | 6.741 | -0.998 | 1329 | 1329 | 42.6 | 1 | 1329 | No intron | D01 | 57651932 | 57653260 | + | 1329 | E.R. |
| Gh_D01G2041 | NA | NA | 438 | 50.042 | -4 | 6.103 | -1.181 | 1317 | 1317 | 38.5 | 1 | 1317 | No intron | D01 | 59409263 | 59410579 | + | 1317 | plas |
| Gh_D02G0706 | NA | NA | 449 | 51.609 | -3 | 6.288 | -1.132 | 1350 | 1350 | 43.2 | 1 | 1350 | No intron | D02 | 10084987 | 10086336 | - | 1350 | E.R. |
| Gh_D04G1685 | NA | NA | 251 | 29.515 | 11.5 | 9.187 | -0.823 | 756 | 756 | 41.9 | 1 | 756 | No intron | D04 | 48986119 | 48986874 | + | 756 | nucl |
| Gh_D04G1686 | NA | NA | 416 | 47.47 | 1.5 | 6.964 | -0.733 | 1251 | 1251 | 42.5 | 1 | 1251 | No intron | D04 | 48987721 | 48988971 | + | 1251 | nucl |
| Gh_D04G2016 | NA | NA | 416 | 47.317 | -1.5 | 6.107 | -0.681 | 1251 | 1251 | 42 | 1 | 1251 | No intron | D04 | 981 | 2231 | + | 1251 | nucl |
| Gh_D07G1233 | NA | NA | 409 | 46.62 | -5.5 | 5.585 | -0.984 | 1230 | 1230 | 43.8 | 1 | 1230 | No intron | D07 | 19017092 | 19018321 | - | 1230 | nucl |
| Gh_D09G2443 | NA | NA | 482 | 54.483 | -2 | 6.364 | -1.075 | 1449 | 1449 | 45.4 | 1 | 1449 | No intron | D09 | 70439 | 71887 | - | 1449 | plas |
| Gh_A01G1153 | PTL | Trihelix transcription factor PTL | 527 | 60.073 | 8 | 7.464 | -0.898 | 1584 | 1584 | 44.6 | 2 | 792 | 543 | A01 | 51004932 | 51007058 | + | 2127 | plas |
| Gh_A01G1784 | PTL | Trihelix transcription factor PTL | 616 | 70.629 | 14 | 7.518 | -0.684 | 1851 | 1851 | 41.1 | 2 | 925.5 | 580 | A01 | 97566016 | 97568446 | + | 2431 | nucl |
| Gh_A05G2476 | PTL | Trihelix transcription factor PTL | 420 | 48.52 | 0 | 6.537 | -0.975 | 1263 | 1263 | 45.9 | 2 | 631.5 | 683 | A05 | 32398207 | 32400152 | + | 1946 | nucl |

| | | | | | | | | | | | | | | | | | | | |
|---|---|---|---|---|---|---|---|---|---|---|---|---|---|---|---|---|---|---|---|
| Gh_A13G0101 | PTL | Trihelix transcription factor PTL | 374 | 43.33 | 6 | 8.099 | -0.92 | 1125 | 1125 | 42.1 | 2 | 562.5 | 510 | A13 | 1192150 | 1193784 | + | 1635 | plas |
| Gh_D01G1259 | PTL | Trihelix transcription factor PTL | 305 | 34.949 | 2.5 | 7.424 | NA | 918 | 918 | 43.1 | 1 | 918 | No intron | D01 | 31865043 | 31865960 | + | 918 | nucl |
| Gh_D01G2026 | PTL | Trihelix transcription factor PTL | 616 | 70.552 | 15 | 7.63 | -0.703 | 1851 | 1851 | 41.1 | 2 | 925.5 | 804 | D01 | 59227031 | 59229685 | + | 2655 | E.R. |
| Gh_D05G2748 | PTL | Trihelix transcription factor PTL | 420 | 48.492 | 0 | 6.535 | -0.965 | 1263 | 1263 | 46.5 | 2 | 631.5 | 646 | D05 | 29672795 | 29674703 | + | 1909 | nucl |
| Gh_D13G0116 | PTL | Trihelix transcription factor PTL | 374 | 43.241 | 6 | 7.814 | -0.951 | 1125 | 1125 | 41.8 | 2 | 562.5 | 430 | D13 | 1161142 | 1162696 | + | 1555 | plas |
| Gh_A11G2872 | pyrH | Uridylate kinase | 481 | 52.683 | 3 | 6.872 | -0.409 | 1446 | 1446 | 48.9 | 7 | 206.6 | 321 | A11 | 92666984 | 92670355 | - | 3372 | plas |
| Gh_D11G3256 | pyrH | Uridylate kinase | 482 | 52.794 | 5 | 7.082 | -0.411 | 1449 | 1449 | 49.2 | 7 | 207 | 340.3 | D11 | 65589386 | 65592876 | - | 3491 | plas |
| Gh_A03G0459 | rnj | Ribonuclease J | 861 | 95.287 | 15 | 7.905 | -0.385 | 2586 | 2586 | 42.3 | 17 | 152.1 | 363.1 | A03 | 10079748 | 10088143 | - | 8396 | nucl |
| Gh_A07G0459 | rnj | Ribonuclease J | 884 | 98.18 | 22.5 | 8.677 | -0.364 | 2655 | 2655 | 42.8 | 17 | 156.2 | 285.4 | A07 | 5916073 | 5923294 | - | 7222 | nucl |
| Gh_D03G1080 | rnj | Ribonuclease J | 860 | 95.39 | 16 | 8.061 | -0.386 | 2583 | 2583 | 42.3 | 17 | 151.9 | 363.3 | D03 | 36085121 | 36093515 | + | 8395 | nucl |
| Gh_D07G0523 | rnj | Ribonuclease J | 884 | 98.266 | 21.5 | 8.675 | -0.359 | 2655 | 2655 | 42.7 | 17 | 156.2 | 298.4 | D07 | 5913650 | 5921079 | - | 7430 | nucl |
| | | | | | | | | | | | | | | | | | | | |
| B. physiochemical properties of *G. raimondii* proteins encoding the *TH* genes | | | | | | | | | | | | | | | | | | | |
| **Gene ID** | **Gene Name** | **Description** | **Protein Length (aa)** | **Molecular Weight (kDa)** | **Charge** | **Isoelectric Point** | **Grand Average of Hydropathy** | **Transcript Length (bp)** | **CDS Length (bp)** | **CDS GC Content (%)** | **Exon Number** | **Mean Exon Length (bp)** | **Mean Intron Length (bp)** | **Chromosome** | **Start** | **End** | **Strand** | **Length (bp)** | **sub cellular localization** |
| Gorai.001G004200 | ASIL1 | Trihelix transcription factor ASIL1 | 350 | 41.218 | -36.5 | 4.227 | -1.261 | 1672 | 1053 | 43.8 | 2 | 836 | 1122 | Chr01 | 411988 | 414793 | + | 2806 | nucl |
| Gorai.010G228000 | ASIL1 | Trihelix transcription factor ASIL1 | 399 | 46.004 | -31 | 4.369 | -1.277 | 1959 | 1200 | 43.2 | 2 | 979.5 | 394 | Chr10 | 59987994 | 59990346 | + | 2353 | nucl |
| Gorai.011G076900 | ASIL1 | Trihelix transcription factor ASIL1 | 400 | 45.702 | -31 | 4.326 | -1.138 | 2285 | 1203 | 43.6 | 2 | 1142.5 | 103 | Chr11 | 7585603 | 7587990 | - | 2388 | nucl |
| Gorai.001G233700 | ASIL2 | Trihelix transcription factor ASIL2 | 267 | 30.91 | -1 | 6.281 | -1.021 | 1643 | 804 | 48.9 | 1 | 1643 | No intron | Chr01 | 47097528 | 47099170 | + | 1643 | E.R. |
| Gorai.004G167100 | ASIL2 | Trihelix transcription factor ASIL2 | 374 | 41.649 | 14 | 9.945 | -0.942 | 1873 | 1125 | 51.9 | 2 | 936.5 | 373 | Chr04 | 46413423 | 46415803 | + | 2381 | E.R. |
| Gorai.006G098600 | ASIL2 | Trihelix transcription factor ASIL2 | 357 | 41.689 | -10.5 | 4.933 | -1.244 | 2251 | 1074 | 41.2 | 1 | 2251 | No intron | Chr06 | 33896937 | 33899187 | - | 2251 | mito |
| Gorai.007G154900 | ASIL2 | Trihelix transcription factor ASIL2 | 278 | 32.255 | 10 | 9.664 | -0.844 | 1337 | 837 | 47.7 | 1 | 1337 | No intron | Chr07 | 13238661 | 13239997 | + | 1337 | E.R. |
| Gorai.008G230600 | ASIL2 | Trihelix transcription factor ASIL2 | 424 | 47.625 | 7.5 | 8.347 | -0.815 | 2336 | 1275 | 43.6 | 1 | 2336 | No intron | Chr08 | 51695916 | 51698251 | + | 2336 | E.R. |

| | | | | | | | | | | | | | | | | | | |
|---|---|---|---|---|---|---|---|---|---|---|---|---|---|---|---|---|---|---|
| Gorai.009G133300 | ASIL2 | Trihelix transcription factor ASIL2 | 376 | 43.507 | -15.5 | 4.736 | -1.141 | 1660 | 1131 | 41.8 | 3 | 553.3 | 184.5 | Chr09 | 10051344 | 10053372 | + | 2029 | mito |
| Gorai.009G144800 | ASIL2 | Trihelix transcription factor ASIL2 | 387 | 42.337 | 12 | 9.967 | -0.875 | 1164 | 1164 | 49.8 | 1 | 1164 | No intron | Chr09 | 10963323 | 10964486 | + | 1164 | E.R. |
| Gorai.009G162200 | ASIL2 | Trihelix transcription factor ASIL2 | 76 | 9.066 | -1 | 5.112 | -0.983 | 310 | 231 | 48.5 | 1 | 310 | No intron | Chr09 | 12455839 | 12456148 | + | 310 | nucl |
| Gorai.010G235700 | ASIL2 | Trihelix transcription factor ASIL2 | 453 | 50.357 | 16 | 9.997 | -0.972 | 1832 | 1362 | 45.9 | 1 | 1832 | No intron | Chr10 | 60607242 | 60609073 | - | 1832 | plas |
| Gorai.011G039600 | ASIL2 | Trihelix transcription factor ASIL2 | 303 | 33.982 | 6.5 | 9.466 | -0.765 | 2162 | 912 | 48.8 | 3 | 720.7 | 1298 | Chr11 | 2958973 | 2964055 | + | 5083 | E.R. |
| Gorai.011G216400 | ASIL2 | Trihelix transcription factor ASIL2 | 372 | 40.798 | 14.5 | 10.035 | -0.858 | 1709 | 1119 | 47.9 | 2 | 854.5 | 1044 | Chr11 | 52112277 | 52115029 | + | 2753 | plas |
| Gorai.012G018400 | ASIL2 | Trihelix transcription factor ASIL2 | 350 | 38.772 | 17 | 10.235 | -0.883 | 1775 | 1053 | 45.1 | 2 | 887.5 | 1194 | Chr12 | 2160832 | 2163800 | + | 2969 | plas |
| Gorai.013G017000 | ASIL2 | Trihelix transcription factor ASIL2 | 126 | 14.497 | -1 | 6.118 | -0.96 | 381 | 381 | 46.5 | 2 | 190.5 | 115 | Chr13 | 1168932 | 1169427 | - | 496 | nucl |
| Gorai.002G021600 | ASR3 | Trihelix transcription factor ASR3 | 363 | 39.636 | -4.5 | 5.375 | -0.715 | 1669 | 1092 | 54.9 | 3 | 556.3 | 766.5 | Chr02 | 1495377 | 1498578 | + | 3202 | nucl |
| Gorai.002G169000 | ASR3 | Trihelix transcription factor ASR3 | 303 | 34.88 | 12 | 9.158 | -1.083 | 1349 | 912 | 48.1 | 2 | 674.5 | 791 | Chr02 | 42103003 | 42105142 | - | 2140 | plas |
| Gorai.009G150000 | ASR3 | Trihelix transcription factor ASR3 | 349 | 38.92 | 7.5 | 9.206 | -0.781 | 1294 | 1050 | 46.6 | 2 | 647 | 383 | Chr09 | 11392324 | 11394031 | + | 1708 | mito |
| Gorai.010G171500 | ASR3 | Trihelix transcription factor ASR3 | 346 | 38.701 | 5 | 8.576 | -0.769 | 1104 | 1041 | 46.1 | 3 | 368 | 291.5 | Chr10 | 49861250 | 49863428 | - | 2179 | nucl |
| Gorai.011G050400 | ASR3 | Trihelix transcription factor ASR3 | 350 | 39.415 | 7.5 | 8.999 | -0.963 | 1697 | 1053 | 44.6 | 2 | 848.5 | 209 | Chr11 | 3936985 | 3939064 | - | 2080 | plas |
| Gorai.007G283800 | At5g28300 | Trihelix transcription factor GTL2 | 667 | 75.558 | 0.5 | 6.544 | -0.971 | 2595 | 2004 | 42.2 | 2 | 1297.5 | 362 | Chr07 | 48589907 | 48592863 | + | 2957 | plas |
| Gorai.002G190600 | GT-1 | Trihelix transcription factor GT-1 | 373 | 42.714 | 2 | 6.754 | -0.702 | 1395 | 1122 | 45 | 5 | 279 | 666.3 | Chr02 | 51467831 | 51472546 | + | 4716 | plas |
| Gorai.008G163200 | GT-1 | Trihelix transcription factor GT-1 | 381 | 43.74 | 2 | 6.726 | -0.821 | 2036 | 1146 | 45.5 | 5 | 407.2 | 779.5 | Chr08 | 43096020 | 43101173 | + | 5154 | plas |
| Gorai.009G333800 | GT-1 | Trihelix transcription factor GT-1 | 393 | 45.024 | 2.5 | 6.738 | -0.958 | 2099 | 1182 | 46.9 | 6 | 349.8 | 865.2 | Chr09 | 34933350 | 34939785 | - | 6436 | plas |
| Gorai.001G020300 | GT-2 | Trihelix transcription factor GT-2 | 595 | 67.079 | 10.5 | 7.183 | -0.901 | 2211 | 1788 | 40.4 | 2 | 1105.5 | 87 | Chr01 | 1894762 | 1897190 | + | 2429 | plas |
| Gorai.002G231500 | GT-2 | Trihelix transcription factor GT-2 | 475 | 53.739 | 12.5 | 9.191 | -1.013 | 2050 | 1428 | 42.3 | 2 | 1025 | 99 | Chr02 | 59100465 | 59102613 | + | 2149 | plas |
| Gorai.003G072300 | GT-2 | Trihelix transcription factor GT-2 | 666 | 73.079 | 9 | 8.417 | -0.825 | 3405 | 2001 | 48 | 4 | 851.3 | 227.3 | Chr03 | 17166033 | 17170119 | + | 4087 | plas |
| Gorai.008G099700 | GT-2 | Trihelix transcription factor GT-2 | 465 | 53.659 | -1 | 6.362 | -1.019 | 2804 | 1398 | 41.9 | 2 | 1402 | 324 | Chr08 | 28847706 | 28850833 | + | 3128 | nucl |
| Gorai.008G262400 | GT-2 | Trihelix transcription factor GT-2 | 792 | 85.776 | -9.5 | 5.666 | -0.783 | 3975 | 2379 | 50 | 3 | 1325 | 414 | Chr08 | 54272520 | 54277322 | - | 4803 | plas |
| Gorai.009G253700 | GT-2 | Trihelix transcription factor GT-2 | 596 | 65.897 | 6 | 7.622 | -0.961 | 2876 | 1791 | 48.7 | 2 | 1438 | 570 | Chr09 | 20820875 | 20824320 | + | 3446 | plas |
| Gorai.009G253900 | GT-2 | Trihelix transcription factor GT-2 | 569 | 63.891 | 0 | 6.526 | -1.021 | 2770 | 1710 | 46.5 | 2 | 1385 | 652 | Chr09 | 20830043 | 20833464 | - | 3422 | plas |

| | | | | | | | | | | | | | | | | | | | |
|---|---|---|---|---|---|---|---|---|---|---|---|---|---|---|---|---|---|---|---|
| Gorai.010G096900 | GT-2 | Trihelix transcription factor GT-2 | 458 | 51.938 | 10.5 | 8.119 | -0.787 | 2269 | 1377 | 41.7 | 2 | 1134.5 | 70 | Chr10 | 16475249 | 16477587 | + | 2339 | E.R. |
| Gorai.010G248600 | GT-2 | Trihelix transcription factor GT-2 | 409 | 46.079 | -1.5 | 6.335 | -0.849 | 1876 | 1230 | 46 | 2 | 938 | 2175 | Chr10 | 61660612 | 61664691 | - | 4080 | nucl |
| Gorai.006G183000 | GT-3A | Trihelix transcription factor GT-3a | 285 | 34.349 | 4 | 7.665 | -1.319 | 1459 | 858 | 45.6 | 2 | 729.5 | 153 | Chr06 | 44052438 | 44054049 | + | 1612 | vacu |
| Gorai.002G262900 | GT-3B | Trihelix transcription factor GT-3b | 259 | 31.418 | 7 | 8.469 | -1.22 | 950 | 780 | 40.5 | 2 | 475 | 88 | Chr02 | 62335347 | 62336384 | - | 1038 | E.R. |
| Gorai.004G174900 | GT-3B | Trihelix transcription factor GT-3b | 261 | 31.258 | 1 | 6.967 | -1.337 | 1544 | 786 | 48 | 2 | 772 | 815 | Chr04 | 47738397 | 47740755 | - | 2359 | cyto |
| Gorai.012G114700 | GT-3B | Trihelix transcription factor GT-3b | 273 | 32.658 | 6 | 8.335 | -1.252 | 1546 | 822 | 45.6 | 4 | 386.5 | 614.3 | Chr12 | 26465324 | 26468712 | + | 3389 | E.R. |
| Gorai.001G139700 | NA | NA | 409 | 46.836 | -5 | 5.607 | -1.007 | 1933 | 1230 | 43.6 | 3 | 644.3 | 144 | Chr01 | 18657686 | 18659974 | - | 2289 | nucl |
| Gorai.002G231400 | NA | NA | 442 | 50.964 | 3.5 | 6.768 | -1.005 | 2182 | 1329 | 42.5 | 1 | 2182 | No intron | Chr02 | 59082050 | 59084231 | + | 2182 | E.R. |
| Gorai.002G243900 | NA | NA | 437 | 49.886 | -7 | 5.743 | -1.179 | 1752 | 1314 | 38.7 | 2 | 876 | 206 | Chr02 | 60802515 | 60804472 | + | 1958 | plas |
| Gorai.005G079200 | NA | NA | 447 | 51.351 | -2 | 6.363 | -1.119 | 2800 | 1344 | 43 | 1 | 2800 | No intron | Chr05 | 9115344 | 9118143 | - | 2800 | plas |
| Gorai.006G159000 | NA | NA | 482 | 54.496 | -2 | 6.364 | -1.076 | 2179 | 1449 | 45.7 | 2 | 1089.5 | 85 | Chr06 | 41839736 | 41841999 | - | 2264 | plas |
| Gorai.012G159500 | NA | NA | 807 | 92.341 | 1 | 6.625 | -0.625 | 2566 | 2424 | 41.7 | 3 | 855.3 | 333.5 | Chr12 | 33219555 | 33222787 | + | 3233 | nucl |
| Gorai.002G159600 | PTL | Trihelix transcription factor PTL | 527 | 60.006 | 8.5 | 7.645 | -0.902 | 2236 | 1584 | 44.3 | 2 | 1118 | 543 | Chr02 | 35260099 | 35262877 | + | 2779 | plas |
| Gorai.002G242000 | PTL | Trihelix transcription factor PTL | 617 | 70.699 | 14 | 7.528 | -0.713 | 2113 | 1854 | 41 | 2 | 1056.5 | 573 | Chr02 | 60634354 | 60637102 | + | 2749 | E.R. |
| Gorai.009G304600 | PTL | Trihelix transcription factor PTL | 420 | 48.58 | -1 | 6.347 | -0.978 | 1846 | 1263 | 46.5 | 2 | 923 | 673 | Chr09 | 27501394 | 27503912 | + | 2519 | nucl |
| Gorai.013G013400 | PTL | Trihelix transcription factor PTL | 374 | 43.264 | 6 | 7.814 | -0.945 | 1249 | 1125 | 41.8 | 2 | 624.5 | 430 | Chr13 | 898580 | 900258 | + | 1679 | plas |
| Gorai.007G371500 | pyrH | Uridylate kinase | 481 | 52.741 | 5 | 7.082 | -0.421 | 2000 | 1446 | 49.2 | 7 | 285.7 | 331.5 | Chr07 | 60441769 | 60445773 | - | 4005 | plas |
| Gorai.001G059600 | rnj | Ribonuclease J | 884 | 98.33 | 21.5 | 8.675 | -0.367 | 3409 | 2655 | 42.6 | 18 | 189.4 | 282.1 | Chr01 | 5891031 | 5899234 | - | 8204 | nucl |
| Gorai.003G119600 | rnj | Ribonuclease J | 860 | 95.351 | 16.5 | 8.064 | -0.392 | 3482 | 2583 | 42.4 | 17 | 204.8 | 361.7 | Chr03 | 35963308 | 35972576 | + | 9269 | nucl |
| | | | | | | | | | | | | | | | | | | | |
| C. Physiochemical properties of *G. arboreum* proteins encoding the *TH* genes | | | | | | | | | | | | | | | | | | | |
| **Protein ID** | **Gene Name** | **Description** | **Protein Length (aa)** | **Molecular Weight (kDa)** | **Charge** | **Isoelectric Point** | **Grand Average of Hydropathy** | **Transcript Length (bp)** | **CDS Length (bp)** | **CDS GC Content (%)** | **Exon Number** | **Mean Exon Length (bp)** | **Mean Intron Length (bp)** | **Chromosome** | **Start** | **End** | **Strand** | **Length (bp)** | **sub cellular localization** |

| | | | | | | | | | | | | | | | | | |
|---|---|---|---|---|---|---|---|---|---|---|---|---|---|---|---|---|---|
| Ga06G2300 | ASIL1 | Trihelix transcription factor ASIL1 | 399 | 46.003 | -31.5 | 4.375 | -1.287 | 1200 | 1200 | 42.9 | 1 | 1200 | No intron | Chr06 | 129248345 | 129249544 | + | 1200 | E.R. |
| Ga07G0077 | ASIL1 | Trihelix transcription factor ASIL1 | 349 | 41.083 | -36 | 4.25 | -1.244 | 1050 | 1050 | 43 | 1 | 1050 | No intron | Chr07 | 852398 | 853447 | - | 1050 | nucl |
| Ga10G2247 | ASIL1 | Trihelix transcription factor ASIL1 | 413 | 47.446 | -44.5 | 4.182 | -1.236 | 1242 | 1242 | 43.8 | 1 | 1242 | No intron | Chr10 | 118271147 | 118272388 | - | 1242 | nucl |
| Ga04G1957 | ASIL2 | Trihelix transcription factor ASIL2 | 330 | 36.448 | 18 | 10.311 | -0.896 | 993 | 993 | 45.8 | 3 | 331 | 663 | Chr04 | 96217243 | 96219561 | - | 2319 | plas |
| Ga05G1355 | ASIL2 | Trihelix transcription factor ASIL2 | 247 | 28.703 | -7 | 4.945 | -1.158 | 744 | 744 | 41.4 | 1 | 744 | No intron | Chr05 | 12019629 | 12020372 | + | 744 | nucl |
| Ga05G1471 | ASIL2 | Trihelix transcription factor ASIL2 | 385 | 42.247 | 12 | 9.978 | -0.877 | 1158 | 1158 | 50.1 | 1 | 1158 | No intron | Chr05 | 13015192 | 13016349 | + | 1158 | E.R. |
| Ga06G2490 | ASIL2 | Trihelix transcription factor ASIL2 | 389 | 42.796 | 8 | 9.412 | -0.941 | 1170 | 1170 | 46.7 | 1 | 1170 | No intron | Chr06 | 130681764 | 130682933 | + | 1170 | plas |
| Ga07G2326 | ASIL2 | Trihelix transcription factor ASIL2 | 255 | 29.25 | -3.5 | 5.52 | -0.96 | 768 | 768 | 50.3 | 2 | 384 | 41 | Chr07 | 92085029 | 92085837 | + | 809 | plas |
| Ga08G1666 | ASIL2 | Trihelix transcription factor ASIL2 | 374 | 41.602 | 14 | 9.945 | -0.951 | 1125 | 1125 | 51.9 | 1 | 1125 | No intron | Chr08 | 107077030 | 107078154 | + | 1125 | plas |
| Ga08G1919 | ASIL2 | Trihelix transcription factor ASIL2 | 340 | 37.89 | 5 | 7.986 | -0.583 | 1023 | 1023 | 45.1 | 2 | 511.5 | 157 | Chr08 | 114264796 | 114265975 | + | 1180 | nucl |
| Ga09G0987 | ASIL2 | Trihelix transcription factor ASIL2 | 357 | 41.444 | -11 | 4.868 | -1.224 | 1074 | 1074 | 41.2 | 1 | 1074 | No intron | Chr09 | 62016124 | 62017197 | - | 1074 | mito |
| Ga10G0825 | ASIL2 | Trihelix transcription factor ASIL2 | 357 | 39.128 | 16.5 | 10.26 | -0.771 | 1074 | 1074 | 48.2 | 3 | 358 | 676 | Chr10 | 17035592 | 17038017 | - | 2426 | plas |
| Ga10G2693 | ASIL2 | Trihelix transcription factor ASIL2 | 303 | 33.951 | 6.5 | 9.466 | -0.758 | 912 | 912 | 48.8 | 2 | 456 | 1953 | Chr10 | 126055070 | 126057934 | - | 2865 | E.R. |
| Ga11G2584 | ASIL2 | Trihelix transcription factor ASIL2 | 247 | 29.07 | -3 | 5.521 | -0.954 | 744 | 744 | 46.9 | 3 | 248 | 45 | Chr11 | 107849624 | 107850457 | - | 834 | E.R. |
| Ga12G0665 | ASIL2 | Trihelix transcription factor ASIL2 | 425 | 47.679 | 9 | 8.723 | -0.803 | 1278 | 1278 | 43.3 | 1 | 1278 | No intron | Chr12 | 6007792 | 6009069 | - | 1278 | E.R. |
| Ga01G0227 | ASR3 | Trihelix transcription factor ASR3 | 363 | 39.625 | -4 | 5.382 | -0.665 | 1092 | 1092 | 54.4 | 3 | 364 | 755.5 | Chr01 | 1704907 | 1707509 | + | 2603 | nucl |
| Ga05G1525 | ASR3 | Trihelix transcription factor ASR3 | 349 | 38.917 | 7.5 | 9.211 | -0.773 | 1050 | 1050 | 46.6 | 2 | 525 | 344 | Chr05 | 13512508 | 13513901 | + | 1394 | mito |
| Ga06G1726 | ASR3 | Trihelix transcription factor ASR3 | 363 | 40.24 | 5 | 8.575 | -0.643 | 1092 | 1092 | 45.5 | 3 | 364 | 355 | Chr06 | 113056919 | 113058720 | - | 1802 | E.R. |
| Ga10G2592 | ASR3 | Trihelix transcription factor ASR3 | 347 | 39.109 | 5.5 | 8.308 | -0.962 | 1044 | 1044 | 44.2 | 2 | 522 | 222 | Chr10 | 124946025 | 124947290 | + | 1266 | plas |
| Ga14G2061 | ASR3 | Trihelix transcription factor ASR3 | 306 | 35.259 | 10.5 | 8.878 | -1.083 | 921 | 921 | 47.7 | 2 | 460.5 | 794 | tig00017379 | 40770 | 42484 | - | 1715 | plas |
| Ga04G0220 | At5g28300 | Trihelix transcription factor GTL2 | 416 | 47.232 | -0.5 | 6.381 | -0.667 | 1251 | 1251 | 42.2 | 1 | 1251 | No intron | Chr04 | 2827610 | 2828860 | - | 1251 | nucl |
| Ga11G1013 | At5g28300 | Trihelix transcription factor GTL2 | 681 | 77.152 | 3 | 6.679 | -1.007 | 2046 | 2046 | 41.9 | 2 | 1023 | 367 | Chr11 | 17799214 | 17801626 | - | 2413 | plas |
| Ga02G0888 | GT-1 | Trihelix transcription factor GT-1 | 365 | 41.861 | 7 | 7.783 | -0.753 | 1098 | 1098 | 45 | 5 | 219.6 | 656 | Chr02 | 59007196 | 59010917 | + | 3722 | E.R. |
| Ga05G3436 | GT-1 | Trihelix transcription factor GT-1 | 375 | 43.014 | 2.5 | 6.738 | -0.898 | 1128 | 1128 | 46.4 | 6 | 188 | 576.8 | Chr05 | 51020086 | 51024097 | - | 4012 | plas |

| | | | | | | | | | | | | | | | | | | |
|---|---|---|---|---|---|---|---|---|---|---|---|---|---|---|---|---|---|---|
| Ga12G1381 | GT-1 | Trihelix transcription factor GT-1 | 362 | 41.413 | 2.5 | 6.8 | -0.733 | 1089 | 1089 | 45.5 | 6 | 181.5 | 624.8 | Chr12 | 19122148 | 19126360 | - | 4213 | E.R. |
| Ga01G1847 | GT-2 | Trihelix transcription factor GT-2 | 669 | 73.657 | 10 | 8.545 | -0.832 | 2010 | 2010 | 48 | 3 | 670 | 265 | Chr01 | 87149752 | 87152291 | - | 2540 | plas |
| Ga02G1480 | GT-2 | Trihelix transcription factor GT-2 | 492 | 55.992 | 12 | 9.046 | -0.969 | 1479 | 1479 | 41.9 | 2 | 739.5 | 96 | Chr02 | 94744692 | 94746266 | + | 1575 | E.R. |
| Ga05G2566 | GT-2 | Trihelix transcription factor GT-2 | 583 | 64.332 | 3.5 | 7.1 | -0.962 | 1752 | 1752 | 49 | 3 | 584 | 308 | Chr05 | 25143255 | 25145622 | + | 2368 | plas |
| Ga05G2567 | GT-2 | Trihelix transcription factor GT-2 | 575 | 64.387 | -3.5 | 6.039 | -0.97 | 1728 | 1728 | 46.5 | 2 | 864 | 644 | Chr05 | 25148973 | 25151344 | - | 2372 | plas |
| Ga06G0929 | GT-2 | Trihelix transcription factor GT-2 | 458 | 51.847 | 9 | 7.948 | -0.821 | 1377 | 1377 | 42 | 2 | 688.5 | 71 | Chr06 | 26013218 | 26014665 | + | 1448 | E.R. |
| Ga06G2330 | GT-2 | Trihelix transcription factor GT-2 | 409 | 46.015 | -5.5 | 5.688 | -0.846 | 1230 | 1230 | 45.9 | 2 | 615 | 2166 | Chr06 | 129530197 | 129533592 | - | 3396 | nucl |
| Ga07G0213 | GT-2 | Trihelix transcription factor GT-2 | 593 | 67.038 | 8.5 | 7.018 | -0.897 | 1782 | 1782 | 40.3 | 2 | 891 | 87 | Chr07 | 2294887 | 2296755 | + | 1869 | plas |
| Ga12G0358 | GT-2 | Trihelix transcription factor GT-2 | 790 | 85.798 | -10 | 5.654 | -0.785 | 2373 | 2373 | 49.3 | 3 | 791 | 419 | Chr12 | 2989912 | 2993122 | + | 3211 | plas |
| Ga14G2668 | GT-2 | Trihelix transcription factor GT-2 | 464 | 53.59 | -1 | 6.362 | -1.047 | 1395 | 1395 | 41.6 | 2 | 697.5 | 328 | tig00023851 | 68444 | 70166 | + | 1723 | nucl |
| Ga09G1979 | GT-3A | Trihelix transcription factor GT-3a | 288 | 34.659 | 3.5 | 7.634 | -1.346 | 867 | 867 | 45.2 | 2 | 433.5 | 153 | Chr09 | 77765153 | 77766172 | + | 1020 | vacu |
| Ga04G0617 | GT-3B | Trihelix transcription factor GT-3b | 273 | 32.561 | 6.5 | 8.621 | -1.204 | 822 | 822 | 46.4 | 2 | 411 | 92 | Chr04 | 11659275 | 11660188 | - | 914 | E.R. |
| Ga08G1740 | GT-3B | Trihelix transcription factor GT-3b | 261 | 31.288 | 0 | 6.603 | -1.393 | 786 | 786 | 48.2 | 2 | 393 | 714 | Chr08 | 109035774 | 109037273 | - | 1500 | cyto |
| Ga14G0071 | GT-3B | Trihelix transcription factor GT-3b | 259 | 31.631 | 11 | 9.337 | -1.315 | 780 | 780 | 39.6 | 2 | 390 | 86 | tig00000254 | 466751 | 467616 | + | 866 | E.R. |
| Ga04G0221 | GTL1 | Trihelix transcription factor GTL1 | 339 | 39.519 | 7 | 8.272 | -0.755 | 1020 | 1020 | 40.9 | 2 | 510 | 184 | Chr04 | 2829516 | 2830719 | - | 1204 | nucl |
| Ga02G1479 | msantd4 | Myb/SANT-like DNA-binding domain-containing protein 4 | 442 | 50.948 | 4.5 | 6.85 | -1.019 | 1329 | 1329 | 41.6 | 1 | 1329 | No intron | Chr02 | 94727604 | 94728932 | + | 1329 | plas |
| Ga03G0752 | msantd4 | Myb/SANT-like DNA-binding domain-containing protein 4 | 449 | 51.575 | -4.5 | 6.155 | -1.139 | 1350 | 1350 | 42.7 | 1 | 1350 | No intron | Chr03 | 11888742 | 11890091 | - | 1350 | plas |
| Ga02G1613 | NA | NA | 437 | 50.161 | -7 | 5.742 | -1.161 | 1314 | 1314 | 37.9 | 1 | 1314 | No intron | Chr02 | 97260911 | 97262224 | + | 1314 | plas |
| Ga07G1387 | NA | NA | 412 | 46.88 | -2.5 | 6.099 | -1.003 | 1239 | 1239 | 43.6 | 1 | 1239 | No intron | Chr07 | 25232270 | 25233508 | - | 1239 | nucl |
| Ga09G1630 | NA | NA | 482 | 54.438 | -2 | 6.364 | -1.07 | 1449 | 1449 | 45.7 | 1 | 1449 | No intron | Chr09 | 73754518 | 73755966 | - | 1449 | plas |
| Ga01G1572 | PTL | Trihelix transcription factor PTL | 527 | 60.088 | 8 | 7.464 | -0.908 | 1584 | 1584 | 44.6 | 2 | 792 | 543 | Chr01 | 59318921 | 59321047 | + | 2127 | plas |
| Ga02G1596 | PTL | Trihelix transcription factor PTL | 616 | 70.659 | 14 | 7.528 | -0.686 | 1851 | 1851 | 41 | 2 | 925.5 | 581 | Chr02 | 97065229 | 97067660 | + | 2432 | nucl |
| Ga05G3094 | PTL | Trihelix transcription factor PTL | 420 | 48.432 | 1 | 6.736 | -0.969 | 1263 | 1263 | 46.2 | 2 | 631.5 | 684 | Chr05 | 35580506 | 35582452 | + | 1947 | nucl |

| | | | | | | | | | | | | | | | | | | |
|---|---|---|---|---|---|---|---|---|---|---|---|---|---|---|---|---|---|---|
| Ga13G0125 | PTL | Trihelix transcription factor PTL | 374 | 43.447 | 6 | 8.215 | -0.944 | 1125 | 1125 | 41.6 | 2 | 562.5 | 521 | Chr13 | 1320994 | 1322639 | + | 1646 | plas |
| Ga11G0095 | pyrH | Uridylate kinase | 480 | 52.636 | 3.5 | 6.91 | -0.412 | 1443 | 1443 | 48.6 | 7 | 206.1 | 321.3 | Chr11 | 646428 | 649798 | + | 3371 | plas |
| Ga01G2125 | rnj | Ribonuclease J | 860 | 95.308 | 16 | 8.059 | -0.389 | 2583 | 2583 | 42.4 | 17 | 151.9 | 363.7 | Chr01 | 100753815 | 100762216 | - | 8402 | nucl |
| Ga07G0614 | rnj | Ribonuclease J | 884 | 98.219 | 22.5 | 8.677 | -0.359 | 2655 | 2655 | 42.9 | 17 | 156.2 | 285.6 | Chr07 | 6755879 | 6763102 | - | 7224 | nucl |
